# Exploratory identification of candidate SNP markers associated with recurrent clinical mastitis in Holstein cattle

**DOI:** 10.1371/journal.pone.0355230

**Published:** 2026-07-30

**Authors:** Shiho Miyata, Lijie Fan, Masahiko Ito, Jun Kambe, Satoshi Takemoto, Chunmei Li, Yuki Yamamoto, Kentaro Nagaoka

**Affiliations:** 1 Laboratory of Veterinary Physiology, Department of Veterinary Medicine, Tokyo University of Agriculture and Technology, Tokyo, Japan; 2 Department of Virology and Parasitology, Hamamatsu University School of Medicine, Shizuoka, Japan; 3 Central Research Institute for Feed and Livestock, ZEN-NOH (National Federation of Agricultural Cooperative Associations), Ibaraki, Japan; 4 College of Animal Science and Technology, Nanjing Agricultural University, Nanjing, China; INRAE Centre Val de Loire: Institut National de Recherche pour l’Agriculture l’Alimentation et l’Environnement Centre Val de Loire, FRANCE

## Abstract

Mastitis remains one of the most prevalent and economically significant diseases in dairy cattle worldwide. Although somatic cell count (SCC) is widely used as an indicator for mastitis diagnosis, its physiological variability limits its utility for predicting individual susceptibility. In this study, we aimed to identify genomic markers associated with recurrent clinical mastitis by defining mastitis-susceptible cows as those experiencing three or more episodes within a single lactation. Whole-genome resequencing was conducted on 50 Holstein cows (25 mastitis-susceptible and 25 healthy controls), yielding 536,184 high-quality SNPs after stringent quality control. A genome-wide association analysis identified 86 SNPs surpassing the significance threshold (–log₁₀P > 5.0), and seven candidate SNPs were evaluated in an independent cohort of 100 cows. Notably, the majority of candidate SNPs were localized on the X chromosome, suggesting a potential role for X-linked variation in mastitis immune response and disease resistance. While the candidate marker panel combining SNP1, SNP2, and SNP7 demonstrated moderate sensitivity (47%), its high specificity (98%) highlights its potential utility as a preliminary screening tool for identifying individuals at increased risk of recurrent mastitis. These findings provide a foundation for further functional validation and large-scale replication studies, which are essential for implementing effective genomic selection strategies to mitigate mastitis incidence in dairy herds.

## Introduction

Mastitis is one of the most prevalent diseases affecting dairy cattle worldwide, leading to substantial economic losses due to reduced milk yield, premature culling, and increased veterinary costs [1]. Developing effective strategies for prediction and prevention is crucial, as the routine use of antibiotics as a first-line treatment not only results in antibiotic residues in milk but also contributes to the emergence of antibiotic resistance in cattle [2]. Although recent advancements in predictive technologies, such as biomarker-based early diagnosis and herd health monitoring systems, have been introduced, these approaches remain underdeveloped and incomplete [3,4].

Genetic improvement strategies in animal breeding have proven highly effective in enhancing traits such as milk production and disease resistance [5]. In particular, advances in animal genomics, including genome-wide association studies (GWAS), have facilitated the identification of quantitative trait loci (QTLs) and genetic markers, such as single nucleotide polymorphisms (SNPs), that influence phenotypes related to milk production and disease susceptibility [6–9]. Additionally, whole-genome sequencing has enabled a comprehensive analysis of genomic variations, including coding, non-coding, and unannotated regions, and has helped identify candidate mutations and genes associated with clinical mastitis and milk production [10,11]. Despite these advancements, reducing mastitis incidence through genomic selection remains challenging. Mastitis is a low-heritability and multifactorial trait, making genetic improvement through conventional selection difficult. Genomic selection approaches may improve selection accuracy for such traits.

Most studies investigating biomarkers for mastitis diagnosis have primarily relied on SCC as a key indicator [12]. When pathogenic microorganisms infect the mammary gland, a significant influx of immune cells, particularly neutrophils, triggers an inflammatory response, leading to an elevated SCC. However, SCC is known to be affected by various physiological factors such as parity, age, stress, and milk production capacity [13–15]. Although SCC measurements are effective for diagnosing mastitis at a given time point, they may be less reliable for assessing an individual cow’s underlying genetic susceptibility to the disease. Some cows that recover from mastitis with initially low SCC levels have been observed to experience recurrent infections in the same or subsequent lactations [16,17]. Moreover, while low SCC is generally considered indicative of an absence of intramammary infection, some studies have identified low SCC levels as a potential risk factor for the subsequent development of clinical mastitis [18–20]. These findings suggest that SCC-based assessments alone may lead to significant errors in evaluating mastitis susceptibility. These limitations highlight the need for alternative approaches to assess underlying susceptibility, and genetic markers such as SNPs may provide a more stable indicator of individual predisposition to mastitis.

In this study, mastitis-susceptible cows were defined as those that experienced three or more episodes of clinical mastitis (one episode includes onset, healing, and recurrence) within the same lactation period. Cows with two episodes were not included in this category, as a stricter definition was applied to identify individuals with a more clearly defined and consistent susceptibility to mastitis. Recurrent mastitis may reflect an underlying genetic susceptibility or persistent host-related factors, such as impaired immune response, which distinguishes it from sporadic single episodes. Cows with recurrent mastitis often have shorter productive lifespans and lower milk yields [21,22]. Although this definition excludes subclinical cases, focusing specifically on cows with repeated relapses within the same lactation allowed us to target a subgroup with a heightened predisposition to mastitis. Whole-genome analysis was conducted to identify genetic markers associated with mastitis susceptibility by comparing cows with recurrent mastitis to a control group that remained mastitis-free for the past two years. Particular attention was given to the chromosomal distribution of candidate SNPs, including potential associations with X-linked regions, which may play a role in immune response and disease resistance. The findings of this study may contribute to a better understanding of the genetic basis of mastitis susceptibility and provide a foundation for further research to support genomic selection strategies in dairy cattle breeding.

## Materials and methods

### Ethics statement

All animal procedures were performed in accordance with relevant institutional and national guidelines for the care and use of experimental animals. This study protocol was approved by the Animal Care Committee of ZEN-NOH Central Research Institute for Feed and Livestock (Approval No. 1601-017).

### Animals

This study population consisted of approximately 400 Holstein dairy cows maintained at the ZEN-NOH Research Farm in Ibaraki, Japan. This study was conducted using a similar experimental design as described in our previous study [18], with modification as described below. All animals were housed in a free-stall barn and received a total mixed ration (TMR) *ad libitum*, with feed offered to allow for approximately 10% refusals. The ration was formulated using NDS Professional software (version 3; RUM&N, Italy) to meet the metabolizable energy and protein requirements of cows producing approximately 40 kg of milk at 80 days in milk (DIM), in accordance with NRC (2001) guidelines (S1 Table). Milking was performed twice daily, in the morning and afternoon (7:00 and 16:00).

**Case definition**

Clinical mastitis was identified based on a combination of diagnostic criteria, including abnormal findings in the California Mastitis TEST (CMT), visible inflammatory signs in the udder, and bacteriological examination of milk samples. Cows were classified as recurrent mastitis when they experienced at least three independent episodes of clinical mastitis within a single lactation period. Each episode was defined as a distinct event involving diagnosis, treatment, and subsequent return to normal lactation. Subclinical mastitis characterized solely by elevated somatic cell count (SCC) without accompanying clinical symptoms were not included in the definition of mastitis in this study due to focusing on clearly defined clinical phenotypes. In addition, no evidence of pathogen transmission between quarters following recovery was observed during the study period. The diagnostic criteria were defined with reference to our previous study [18].

### Sample size and collection

Animals were selected from a herd of approximately 400 Holstein cows based on veterinary health records. Cows were initially grouped according to their mastitis history. Individuals that have experienced three or more clinical mastitis events within a single lactation were assigned to the recurrent mastitis group, whereas cows with no recorded mastitis across at least two consecutive lactations were defined as healthy. To minimize potential confounding effects, cows with the highest parity levels, representing the top 10 percent within the herd, were excluded from the analysis. After this step, the remaining animals were further screened to confirm normal lactation status at the time of sampling. Because the number of recurrent mastitis cows was limited, 25 individuals were included in this group. An equal number of healthy cows were then randomly selected to establish a balanced dataset for whole genome analysis. In addition, a separate validation cohort consisting of 100 cows from the same herd was used for SNP validation. The validation cohort included 57 healthy cows and 43 cows with recurrent mastitis. Milk production records including yield, fat content, protein content and somatic cell count were obtained from the official Japanese milk recording programs operated by the Livestock Improvement Association of Japan, Inc. Milk samples were analyzed using a Milko Scan CombiFoss™ FT+ (Foss Tokyo, Japan) to assess milk composition and somatic cell count.

### DNA sampling and whole genome sequencing

A total of 50 animals were subjected to whole-genome sequencing analysis. Genomic DNA was extracted from 100 µl of whole blood using the Qiagen DNeasy Blood and Tissue Kit (Qiagen, Hilden, Germany) according to the manufacturer’s protocol. The whole-genome sequencing library was prepared using the TruSeq DNA PCR-Free Library Preparation Kit (Illumina, San Diego, USA). Library yield was assessed with the Library Quantification Kit (Clontech), and fragment size distribution was determined using the 2100 Bioanalyzer DNA 1000 chip (Agilent). Sequencing was performed on the NovaSeq 6000 platform (Illumina, San Diego) using 2 × 151 bp paired-end reads. Library preparation and sequencing were carried out at Takara Bio, Inc. (Shiga, Japan).

### Sequence data analysis

Sequence mapping was performed using CLC Genomics Workbench (version 20, CLC Bio, Aarhus, Denmark). Raw sequences were quality-filtered by removing adapter sequences, filtering out low-quality reads (quality score < 0.05), and discarding reads with more than two ambiguous nucleotides. High-quality reads were aligned to the *Bos taurus* reference genome [Genome assembly ARS-UCD1.2, RefSeq accession: GCF_002263795.1] obtained from NCBI. Local realignment was performed with ‘realign-unaligned ends’ set to ‘yes’ and ‘multi-pass realignment’ set to 2 (S2 Table). SNPs were identified using the ‘Basic Ploidy Variant Detection’ tool with a sample ploidy of 2, minimum coverage of 10, minimum count of 2, and a minimum frequency of 35%. Other filter settings were kept at their default values. Variants were annotated based on functional classification—including synonymous and non-synonymous substitutions—and genomic regions (upstream, downstream, exonic, intronic, and untranslated regions).

### Validation of candidate SNPs

Candidate SNP markers for mastitis susceptibility were validated by PCR and Sanger sequencing. Primer pairs for each SNP were designed using CHOPCHOP (https://chopchop.cbu.uib.no/) based on 250 bp flanking sequences (S3 Table). PCR amplification was performed using KOD FX Neo (TOYOBO, Tokyo, Japan), and the quality and size of the amplified DNA were assessed by 2% agarose gel electrophoresis (S1 Fig). PCR products were purified using Exo-SAP-IT Express (Thermo Fisher Scientific, Waltham, USA) and sequenced using the Sanger method (Eurofins Genomics, Tokyo, Japan).

### Statistical analysis

Genome-wide association analysis was performed using a case–control association test implemented in CLC Genomics Workbench (version 20, QIAGEN). For each SNP, allele frequencies between mastitis-susceptible and healthy cows were compared using a chi-square test to evaluate statistical association. Only SNPs that passed stringent quality control filters during variant calling were retained for the association analysis.

Genome-wide significance was defined as –log₁₀(P) > 5 (P < 1 × 10 ⁻ ⁵). Given the relatively small sample size and the exploratory nature of this study, this threshold was used to identify candidate loci for subsequent validation rather than to establish definitive genome-wide significance. In addition, a suggestive significance threshold of P < 1 × 10 ⁻ ⁶ was applied to identify candidate loci, as commonly used in exploratory GWAS with limited sample sizes [23,24].

### Statistical power calculation

Statistical power calculations were performed to provide an approximate estimate of detectable effect sizes under the present sample size. Because reliable estimates of SNP effect sizes for recurrent mastitis susceptibility are currently limited, we used a hypothetical effect size corresponding to approximately 7% of variance explained (R² ≈ 0.07) as an illustrative scenario rather than a precise biological estimate. Power calculations were performed to evaluate whether the present sample size could detect SNP associations under plausible effect-size scenarios. These calculations were used only to provide an approximate indication of the expected statistical power and should not be interpreted as precise estimates.

We converted reported odds ratios (OR) to logistic regression coefficients (b) using the formula:


$$\begin{array}{c} b=\text{ln}\left(\text{OR}\right) \end{array}$$

Minor allele frequencies (MAF), case proportions (*P*), and population prevalence (*K*) were incorporated to calculate genotype and phenotypic variances following established methods. Population prevalence (*K*) was set to 0.15 based on previous studies [25,26]. Nagelkerke’s R^2^ was computed from logistic regression likelihoods and transformed to liability scale R^2^ using the liability threshold model.

The noncentrality parameter (NCP) for each SNP was calculated based on the sample size (n = 50) and liability scale R^2^:


$$\begin{array}{c} \text{NC}{\text{P}}_{\text{i}}=\sqrt{n\times R_{i}^{\text{2}}} \end{array}$$

Assuming independence among SNPs, the total NCP for the polygenic score was derived as the square root of the sum of squared individual SNP NCPs:


$$\begin{array}{c} \text{NC}{\text{P}}_{\text{total}}=\sqrt{\sum_{i=\text{1}}^{\text{7}}\text{NC}{\text{P}}_{\text{i}}^{\text{2}}} \end{array}$$

Statistical power was estimated using the standard normal distribution and adjusted significance thresholds accounting for multiple testing. Specifically, Bonferroni correction for 7 SNPs yielded an adjusted alpha level of 0.00714 (α = 0.05/7). Power calculations incorporated this threshold to provide realistic detection probabilities.

All calculations of coefficients, variances, NCPs, and power were performed using Microsoft Excel (Microsoft 365) with custom formulas and Python (version 3.12) scripts run in Visual Studio Code (version 1.103.0).

Population structure was evaluated using principal component analysis (PCA) based on genome-wide SNP data. PCA was performed using the PCA module implemented in the scikit-learn library in Python. Because all animals originated from a single herd and no clear genetic stratification was observed in the PCA plot, no additional correction for population structure (e.g., inclusion of principal components or a kinship matrix) was applied. Manhattan and quantile-quantile (Q-Q) plots were generated using the CMplot package (https://github.com/YinLiLin/R-CMplot) in R (http://www.r-project.org/). The effects of the seven SNP genotypes on mastitis incidence and milk production traits were initially analyzed using the Kruskal–Wallis test followed by Dunn’s test for multiple comparisons, with statistical significance set at P < 0.05. To provide more rigorous statistical evaluation, GLMM and Poisson mixed-effects model analyses were additionally performed as described below. SNP marker validation involved calculating sensitivity and specificity based on a 2 × 2 contingency table. Sensitivity was defined as A/(A + C) and specificity as B/(B + D), where A = true positives, B = true negatives, C = false negatives, and D = false positives. Marker reliability was assessed using Cohen’s kappa coefficient, interpreted as follows: fair (0.2–0.4), moderate (0.4–0.6), substantial (0.6–0.8), and excellent (>0.8) [27].

### Significance threshold for association analysis

A significance threshold of –log₁₀(P) > 5.0 (P < 1 × 10 ⁻ ⁵) was applied as an exploratory threshold to prioritize candidate SNPs for subsequent validation. This threshold is less stringent than a strict Bonferroni-corrected genome-wide significance level for 536,184 SNPs (P < 9.3 × 10 ⁻ ⁸; –log₁₀P > 7.03), and was selected to balance detection sensitivity with the exploratory nature of this study.

### Pedigree-based mixed-effects model analysis

To evaluate the potential influence of familial relatedness on the observed associations, sire information was obtained for all 100 cows in the validation cohort. Generalized linear mixed models (GLMMs) were fitted with mastitis status (healthy = 0, mastitis = 1) as the binary response variable, SNP genotype coded as 0, 1, or 2 as a fixed effect, and sire as a random effect. In addition, the number of mastitis episodes was analyzed using a Poisson mixed-effects model with SNP genotype as a fixed effect and sire as a random effect. All mixed-effects model analyses were performed using the lme4 package in R.

## Results

### Sample size validation

For statistical power estimation, each individual SNP was assumed to account for approximately 7% of phenotypic variance on the liability scale (R² ≈ 0.07; S4 Table); however, with the available sample size (n = 50) and Bonferroni correction for 7 SNPs (α = 0.00714), statistical power to detect associations at the single SNP level was low (<20%), as detailed in S4 and S5 Tables.

By aggregating the SNP effects into a polygenic risk score (PRS) and combining their noncentrality parameters, the overall power to detect association increased substantially to approximately 98.5% at the adjusted significance level (S6 Table).

These results suggest that, although individual SNP effects are difficult to detect in isolation given the limited sample size, joint analysis leveraging polygenic effects provides sufficient statistical power.

### Major milk parameters

Milk parameters, including milk yield, fat and protein percent, and somatic cell count (SCC) during the first lactation, were extracted from the monthly examination data of the Milk Recording Program (Table 1).

**Table 1:** Milk characteristics of 50 cows (Healthy:25, Mastitis:25).

	Healthy (25)		Mastitis (25)		
Milk characteristic	Means	SE	Means	SE	*P-*value
**Milk yield (kg/d)**	36.29	1.16	36.08	1.21	0.90
**Fat (%)**	4.14	0.07	4.06	0.08	0.46
**Protein (%)**	3.25	0.03	3.18	0.04	0.23
**SCC (x10**^**4**^ **cells/ml)**	9.34	2.21	43.69	9.12	<0.01
**Parity**	2.68	0.24	2.72	0.25	0.91

Table 1 showing milk characteristics from the Milk Recording Program result of 50 animals. P-value was calculated using a T-test. Cows with three or more episodes of clinical mastitis exhibited significantly higher SCC compared to healthy cows, confirming the phenotypic distinction between the groups.

Bacteriological culture data were available for the 25 mastitis-susceptible cows in the discovery cohort. The most frequently isolated pathogens were coagulase-negative staphylococci (CNS; 15/25 cows), *Escherichia coli* (12/25), *Streptococcus* spp. (12/25), and *Klebsiella* spp. (11/25) (S7 Table). Notably, 21 of 25 cows (84%) experienced mastitis episodes caused by two or more different pathogen species, suggesting that the recurrent mastitis phenotype in these animals may reflect host-related susceptibility rather than persistent single-pathogen infection.

### Identification of candidate SNPs by whole genome resequencing

**Fig 1:**
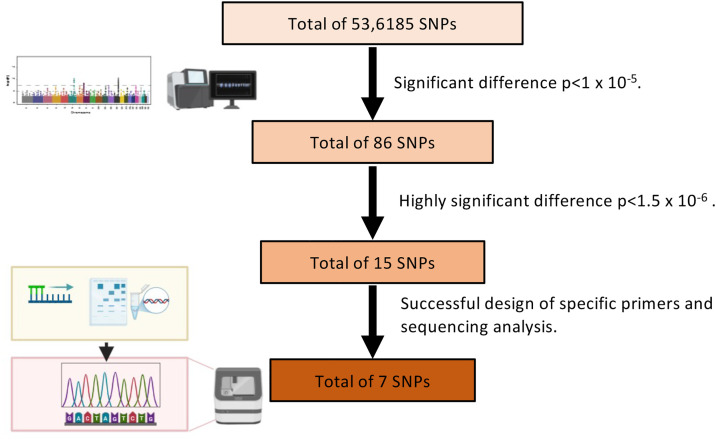
Identification workflow for mastitis susceptibility SNPs. Flowchart summarizing the screening process: starting from a total of 536,185 SNPs, 86 candidate SNPs were selected based on a significance threshold of P < 1 × 10 ⁻ ⁵. Among these, 15 SNPs meeting a more stringent threshold (P < 1.5 × 10 ⁻ ⁶) were prioritized, followed by the successful design of specific primers and sequencing analysis that ultimately yielded 7 candidate SNP markers associated with mastitis susceptibility. A total of 50 animals were used for GWAS.

**Fig 2:**
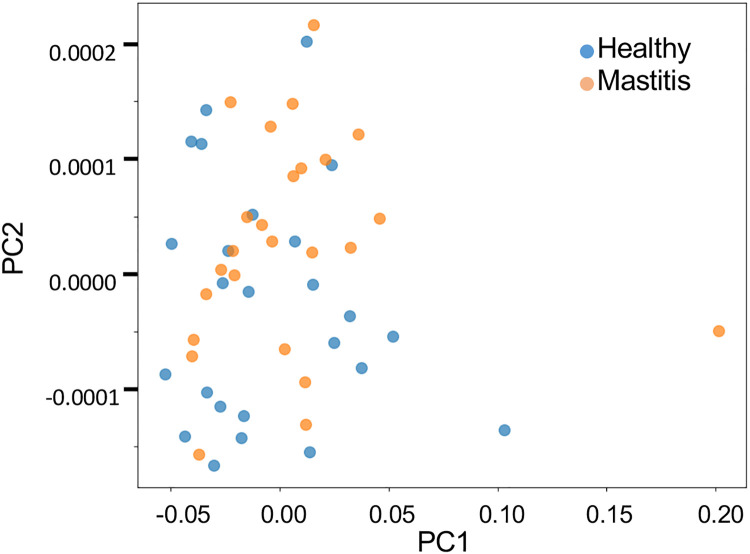
Principal component analysis (PCA) of SNP data. PCA was performed to reduce the dimensionality of SNP data derived from VCF files, enabling visualization of the primary genetic variations between the healthy (blue) and mastitis (orange) groups. The scatter plot displays the first two principal components (PC1 and PC2), with each point representing an individual sample. No clear separation between healthy and mastitis groups was observed.

**Fig 3:**
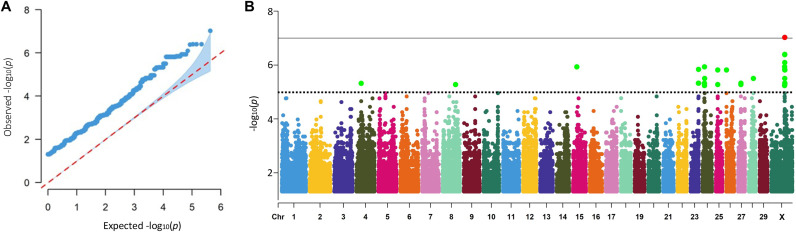
Genome-wide association analysis. (A) Q–Q plot showing the observed –log₁₀(P) values versus the expected distribution under the null hypothesis. (B) Manhattan plot showing genome-wide SNP associations with mastitis susceptibility. Each point represents a SNP. The dashed horizontal line indicates the exploratory significance threshold (–log₁₀P = 5), while the solid horizontal line represents a more stringent genome-wide significance threshold (–log₁₀P = 7). GWAS analysis identified 15 highly associated SNPs for further validation.

**Fig 4:**
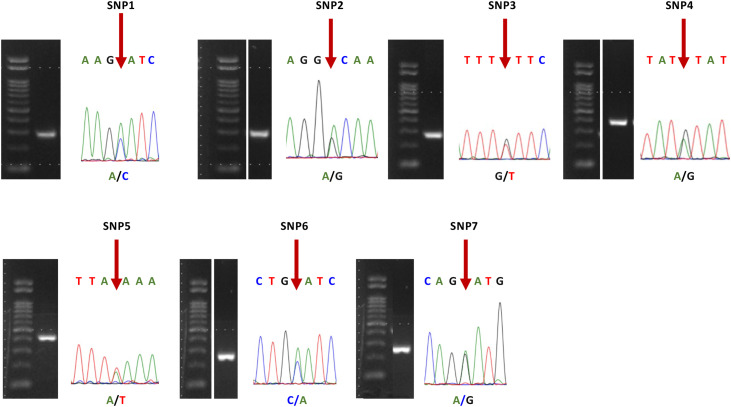
PCR and Sequencing Validation of Candidate SNPs. Representative images of PCR products and corresponding sequencing chromatograms for the seven SNP markers associated with mastitis susceptibility. Allelic variations for each SNP (SNP1–SNP7) are indicated. All selected SNPs were successfully validated by PCR amplification and Sanger sequencing.

Fig 1 presents a flowchart of the screening process. A total of 536,184 SNPs were detected, and PCA analysis revealed no significant differences in SNP distribution between healthy and mastitis groups (Fig 2). The Q-Q plot (Fig 3A) showed that the observed and expected –log₁₀ P-values followed an approximately linear relationship, with an exploratory significance threshold set at –log₁₀ P > 5.0 (P < 1 × 10 ⁻ ⁵). A total of 86 SNPs met this threshold (S8 Table), and their distribution is illustrated in the Manhattan plot (Fig 3B). Among these, 15 SNPs with the highest significance (P < 1.5 × 10 ⁻ ⁶) were selected for further validation. Specific primers were designed, and DNA sequencing via the Sanger method ultimately confirmed 7 candidate SNP markers (Table 2, Fig 4).

**Table 2:** Seven candidate SNP markers associated with mastitis susceptibility were identified by genome-wide association analysis.

SNP ID	SNP1	SNP2	SNP3	SNP4	SNP5	SNP6	SNP7
**Ref SNP**	rs110036757	rs208854668	rs381839020	rs385943123	rs137152785	rs110688314	rs110369890
**Chr**	15	X	X	X	X	X	24
**Position**	25087716	88209479	89239121	89242713	88700655	89067208	12219701
**Reference**	A	A	G	A	A	C	A
**Allele**	C	G	T	G	T	A	G
**Genotype frequency**							
**Ref homo**	40	52	36	36	30	30	50
**Hetero**	52	46	50	50	60	46	48
**Allele homo**	8	2	14	14	10	24	2

Table 2 showing seven candidate SNP markers associated with mastitis susceptibility were identified by genome-wide association analysis. Genotype frequency (%) were calculated. Ref homo:reference homozygote, Hetero:heterozygote, Allele homo:allele homozygote.

**Fig 5:**
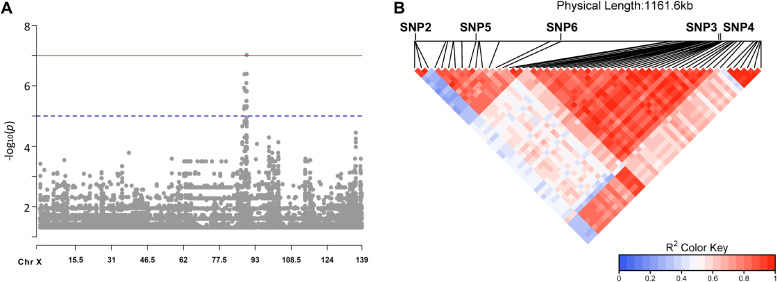
X-Chromosome Association and Linkage Disequilibrium (LD) Analysis. (A) Manhattan plot depicting –log₁₀(P) values for SNPs across the X chromosome, highlighting regions of putative association. (B) LD heatmap showing pairwise r² values among SNPs on the X chromosome within the candidate region. High LD (strong correlations) is indicated by red shading, while blue indicates lower LD. Candidate SNPs were clustered within a specific region of the X chromosome and showed strong linkage disequilibrium.

Notably, most candidate SNPs were located on the X chromosome, clustered within the region between 88,208,181 and 89,369,744 (Fig 5A). Linkage disequilibrium (LD) block analysis revealed that these SNPs were segregated into four blocks; specifically, SNP3 and SNP4, SNP5 and SNP6 were located in another same block, whereas SNP2 was found in a distinct block (Fig 5B).

### Validation of Identified SNPs

The reliability of the seven identified SNP markers for screening mastitis susceptibility was evaluated using DNA sequencing. In total, 100 cows (healthy: n = 57; mastitis: n = 43) were genotyped to assess marker performance. Herd traits, including key milk parameters and parity, are presented in S9 Table to provide background characteristics of the validation population used for SNP validation.

**Fig 6:**
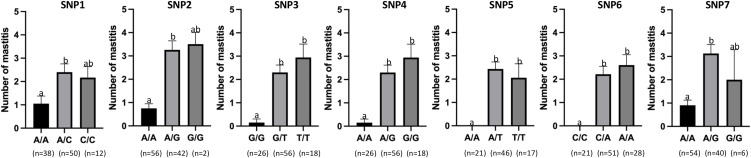
Associations between SNP genotypes and number of mastitis cases. The bar graph shows the mean number of mastitis cases for each genotype. Error bars represent SEM. Different letters indicate significant differences (P < 0.05). SNP genotypes were significantly associated with increased mastitis incidence.

Analysis of the seven SNP genotypes revealed that cows carrying either heterozygous or homozygous mutant alleles consistently exhibited a higher incidence of mastitis compared with those harboring the reference homozygous genotype (Fig 6). These findings suggest an association between these SNPs and mastitis incidence. The impact of these SNPs on major milk traits (milk yield, fat content, and protein content) was also examined (S10 Table). While genotype differences did not significantly affect milk yield or fat content, SNP2 and SNP7 were found to significantly influence milk protein content.

To determine the screening utility of these SNPs, sensitivity, specificity, and Cohen’s kappa coefficient were calculated (Table 3). The corresponding contingency table values for each SNP are provided in S11 Table.

**Table 3:** Seven SNPs result in screening capacity for susceptibility to mastitis.

SNP ID	SNP1	SNP2	SNP3	SNP4	SNP5	SNP6	SNP7
**Ref SNP**	rs110036757	rs208854668	rs381839020	rs385943123	rs137152785	rs110688314	rs110369890
**Sensitivity**	0.79	0.72	0.98	0.98	1.00	1.00	0.67
**Specificity**	0.49	0.77	0.44	0.44	0.37	0.37	0.70
**Kappa**	0.25	0.53	0.34	0.38	0.33	0.33	0.39

Table 3 showing Sensitivity, Specificity, and Kappa coefficient for each of the seven SNP markers for screening mastitis susceptibility based from 100 cows.

Based on kappa coefficient values, SNP2 demonstrated moderate potential as a genomic marker. In addition, SNP combinations were evaluated to maximize specificity and reduce false positives. SNPs included in the combination analysis were selected based on their statistical significance and validation performance. Combinations involving SNP1, SNP2, and/or SNP7 yielded improved screening accuracy (Table 4)

**Table 4:** The combination of SNP results in screening capacity for susceptibility to mastitis.

SNP ID	SNP1 + SNP7	SNP2 + SNP7	SNP1 + SNP2 + SNP7
**Sensitivity**	0.60	0.53	0.47
**Specificity**	0.88	0.95	0.98
**Kappa**	0.50	0.51	0.48

Table 4 showing Sensitivity, Specificity, and Kappa coefficient for combination of two or three SNP markers for screening mastitis susceptibility based from 100 cows.

Among the 43 cows with mastitis, 20 carried heterozygous or homozygous mutations in SNP1, SNP2, and SNP7, corresponding to an approximate sensitivity of 47%. In contrast, only 1 of the 57 healthy cows tested false positive, resulting in an estimated specificity of 98%.

To further assess the potential influence of familial relatedness, sire-adjusted mixed-effects models were performed using the validation cohort. All seven candidate SNPs remained significantly associated with mastitis status after inclusion of sire as a random effect (S12 Table). Similar results were obtained when the number of mastitis episodes was analyzed using a Poisson mixed-effects model, with all candidate SNPs showing significant associations (S12 Table). The estimated variance attributable to sire effects was negligible, suggesting that the observed associations are unlikely to be driven by sire-family structure alone. The distribution of healthy and mastitis cows across major sire families is shown in S2 Fig.

## Discussion

Mastitis is a major economic and animal welfare concern in dairy farming, and identifying individuals susceptible to mastitis is crucial for efficient herd management. However, false positives in traditional susceptibility screening can lead to the unnecessary exclusion of healthy individuals, potentially negatively impacting economic efficiency. In the present study, we focused on individuals with a clear and recurrent disease phenotype by defining mastitis-susceptible cows as those experiencing three or more episodes of clinical mastitis within a single lactation. Using GWAS analysis, we identified seven candidate SNPs associated with mastitis susceptibility in Holstein cows, which were selected for validation based on their statistical significance. These findings should be interpreted as hypothesis-generating rather than evidence of causal relationships.

The predictive quality of the identified SNPs was evaluated using sensitivity, specificity, and kappa coefficient values. Cohen’s kappa coefficient was used to evaluate the agreement between genotype-based classification and actual mastitis status, providing an additional measure of reliability with sensitivity and specificity. All seven SNPs indicated that cows with heterozygous or homozygous mutant genotypes had a higher incidence of mastitis compared to cows with homozygous wild-type genotype. Therefore, cows with mutant allele were considered to have increased susceptibility to recurrent mastitis. The combinations of SNP1, SNP2, and SNP7 showed a sensitivity of 47% and a specificity of 98%. Although the moderate sensitivity suggests that this panel would not identify all susceptible animals, indicating that a substantial proportion of susceptible individuals may be missed, the high specificity demonstrates its potential to minimize false positives. Compared to conventional diagnostic methods such as the Whiteside test, California Mastitis Test (CMT), and SCC-based assessments [28,29], this approach suggests a promising direction for the early identification of cows with genetic predisposition to recurrent mastitis.

The observed association between SNP genotypes and mastitis incidence may reflect underlying genetic susceptibility related to immune response or inflammatory pathways. However, the functional roles of these variants remain unclear, and further studies are required to elucidate the biological mechanisms underlying these associations.

The SNP1, SNP2, and SNP7 markers identified in this study were not located in protein-coding regions; however, SNP1 was found in the intronic region of the neurexophilin and PC-esterase domain family member 4 (NXPE4) gene. In humans, the NXPE4 protein has an Ig-like fold structure and is highly expressed in the colon. Studies have reported reduced NXPE4 expression in colorectal tumors and its association with ulcerative colitis, suggesting a possible role in tumor suppression and inflammation regulation [30–33]. Although the function of the NXPE4 gene in cattle has not yet been reported, these observations imply that it may be worth investigating its potential role in immune regulation and mastitis occurrence. However, these interpretations are based on findings from other species and should be considered speculative in the context of cattle.

In this study, individuals that experienced repeated episodes of mastitis during the same lactation period were defined as mastitis-susceptible. The recurrence of mastitis can also be attributed to persistent infection, where the infection persists even after the resolution of clinical signs, leading to subsequent flare-ups of mastitis [16]. The recurrence event in the previous lactation has also been identified as a risk factor for mastitis in the subsequent lactation [34,35]. These observations indicate that cows with repeated episodes of mastitis in the same lactation are at a higher risk of developing mastitis in the subsequent lactation.

Kurz and colleague conducted a GWAS analysis using a high-density SNP array under the definition of mastitis-susceptible animals as those in which clinical mastitis symptoms, abnormal SCC, and bacterial identification in milk were observed on four or more occasions during the same lactation period, while mastitis-resistant animals were defined as cows that exhibited no clinical signs of mastitis, maintained an SCC below 250,000 cells/ml throughout the period, and showed no evidence of intramammary infection from bacterial cultures [36]. Although 117 candidate SNPs and 27 associated QTLs were identified, their statistical P-values were below 1 × 10^−4^, representing a more lenient significance threshold compared to those used in the current study (P < 1.5 × 10^−6^). It is suggested that the high p-values (low significance) observed in previous studies may be due to the fact that the gene mutations causing clinical mastitis symptoms, abnormal SCC, and intramammary infections differ. In our study, by simply focusing on the recurrence of clinical mastitis, the mastitis-susceptible group was more homogeneous, allowing us to identify variants with lower p-values (highly significant). In addition, recurrent mastitis may be influenced not only by genetic susceptibility but also by factors such as infection persistence and management conditions, which were not fully accounted for in this study. In the future, it will be necessary to continue analyses of gene mutations and expression profiles in both mastitis-susceptible and mastitis-resistant individuals using such an approach.

Our results revealed that the majority of candidate SNP markers putatively associated with mastitis susceptibility are located on the X chromosome, suggesting that X-linked genetic variation may contribute to immune response and disease resistance in dairy cattle. The observed clustering of these markers in a narrow genomic region, characterized by strong linkage disequilibrium and low recombination rates, likely reflects historical selection pressures and the unique inheritance patterns of the X chromosome. Moreover, given the mechanism of X-chromosome inactivation, which balances gene expression between sexes, it is plausible that differential regulation of X-linked genes may influence the inflammatory pathways and cellular responses underlying mastitis. Previous studies have implicated X-linked variants in various traits related to reproductive function and disease resistance [37–39] and recent work by [40] further supports the contribution of the X chromosome to key production traits in dairy cattle. LD analysis on the region between 88208181 and 89369744 (P < 1 × 10^−5^) on the X chromosome revealed four distinct LD blocks. SNP2, which we consider useful in uncovering mastitis susceptible individuals, is located in a separate block from the other candidate SNPs (SNPs 3, 4, 5, and 6), suggesting that these markers are physically close but have different recombination rates and different functional roles. These findings underscore the need for further functional analyses to elucidate the regulatory dynamics of X-linked genes, which could ultimately refine genomic selection strategies aimed at enhancing mastitis resistance. However, the current analytical framework does not account for factors such as sex-specific inheritance, dosage compensation, or X-chromosome inactivation. Therefore, these findings remain speculative, and further studies incorporating these considerations are required to clarify the role of X-linked variation in mastitis susceptibility.

This study has several limitations. The relatively small sample size resulted in limited statistical power for detecting individual SNP associations, as indicated by the post hoc power analysis. This limitation increases the possibility of false positive findings at the SNP level, and therefore the identified associations should be interpreted with caution. In addition, the limited sample size may have reduced the ability to detect variants with small effect sizes. Therefore, the SNPs identified in this study should be considered candidate loci requiring validation in larger and independent populations. The significance threshold used in this study was less stringent than a genome-wide Bonferroni correction, which may increase the risk of false-positive associations. Furthermore, some of the candidate SNPs selected for validation could not be successfully confirmed, likely due to technical limitations such as difficulties in primer design and sequencing quality issues. The focus on clinical mastitis episodes within a single lactation, while excluding subclinical cases to prioritize clearly defined and recurrent clinical phenotypes may have limited the scope of mastitis susceptibility assessment and reduced comparability with previous studies. Although candidate SNP markers were evaluated in an independent cohort, the functional roles of these variants remain uncharacterized. In addition, the primary association analysis did not incorporate a genomic relationship matrix to account for pairwise relatedness among animals. Although supplementary GLMM analyses including sire as a random effect confirmed the robustness of the identified associations, future studies should employ mixed-model approaches with full kinship correction to more rigorously control for population structure. Furthermore, the genomic inflation factor (λ) could not be calculated because the archived association results contained only variants that passed significance filtering rather than the complete genome-wide P-value distribution. The validation cohort was derived from the same herd as the discovery population, which may reduce the independence of validation and limit the generalizability of the findings to broader populations. Future research with larger sample sizes, multiple herds, and comprehensive functional analyses is necessary to validate these findings and enhance the application of genomic selection strategies in reducing mastitis incidence.

This study employed whole-genome resequencing to identify candidate SNP markers associated with recurrent clinical mastitis in Holstein cattle. In conclusion, these findings suggest a possible contribution of X-linked genetic variation to mastitis susceptibility and provide preliminary markers that may aid in the development of genomic selection programs. However, these findings should be interpreted as exploratory, and larger-scale studies and functional validation are needed to fully clarify the biological significance and practical utility of the identified markers for improving mastitis resistance in dairy herds.

## Supporting information

S1 Fig The full-length gel image for seven SNPs. SNP1–SNP7 were included in the analysis. This figure shows the original uncropped and unadjusted gel images corresponding to the processed images presented in the Fig. 4. The electrophoresis image at the bottom left was excluded from the analysis.
(PDF)

S2 Fig Distribution of Healthy and Mastitis Cows by Sire Family. Sire family distribution was examined to evaluate potential familial stratification. The two most represented sire families (12 and 7 offspring, respectively) contained both healthy and mastitis animals, and no obvious clustering of mastitis cases within a single major sire lineage was observed. These observations suggest that the identified associations are unlikely to be explained solely by sire-family structure.
(PDF)

S1 Table Ratio and composition of TMR mixture. 1)Palmitic acid (C16–0). 2)Composition: calcium = 25%; magnesium = 11.6%; copper = 0.16%; zinc = 0.93%; manganese = 0.38%; selenium = 0.001%; iodine = 0.03%; cobalt = 0.023%; vitamin A = 2,500,000 IU; vitamin D = 500,000 IU; vitamin E = 1%; d-biotin = 0.02%.
(XLSX)

S2 Table The mapping parameters for all individuals. Sequencing quality and mapping statistics, including mapped reads, coverage, and quality scores (Q20 and Q30), are shown for each sample.
(XLSX)

S3 Table Primer sequence of seven SNPs. Forward and reverse primer sequences used for genotyping of the seven SNPs.
(XLSX)

S4 Table Required R^2^ for 80% power (n = 50, two-sided). Required R² values to achieve 80% statistical power for a sample size of n = 50 under different significance levels (two-sided test). z₀.₈ = 0.8416. R² values are rounded to three significant figures.
(DOCX)

S5 Table Power analysis for SNP detection under varying effect sizes (n = 50, α = 0.00714). Power analysis for SNP detection under varying effect sizes with n = 50 and α = 0.00714 (two-sided). Power was calculated for different assumed R² values. The non-centrality parameter (NCP) was calculated as √((N × R²)/(1 − R²)).
(DOCX)

S6 Table The summary of statistical calculation power for individual SNPs. Statistical power calculated for each individual SNP analyzed in this study.
(XLSX)

S7 Table Bacteriological culture results for the 25 mastitis-susceptible cows in the discovery cohort. Pathogen species identified at each clinical mastitis episode are shown. CNS, coagulase-negative staphylococci; N.D., not determined; -, no episode recorded.
(XLSX)

S8 Table SNPs identified between healthy and mastitis according to (-log10 *P* value was > 5.0). SNPs identified between healthy and mastitis groups with −log10(P) > 5.0. The table summarizes SNP ID, alleles, chromosomal positions, and associated genes for the detected variants.
(XLSX)

S9 Table Milk characteristics of 100 cows (Healthy;57, Mastitis;43). Table S9 showing milk characteristics from the Milk recording program result of 100 animals. P-value was calculated using T-test.
(XLSX)

S10 Table The effects of SNPs on key milk parameters-milk yield, fat content, and protein content. Effects of SNP genotypes on milk composition parameters. Wild type represents reference homozygotes, whereas mutant includes both heterozygous and mutant homozygous genotypes. Asterisk indicates a statistically significant difference (P < 0.05).
(DOCX)

S11 Table Contingency table values for individual SNP markers and SNP combinations used for screening mastitis susceptibility. Classification was based on a 2 × 2 contingency table, where A = true positives, B = true negatives, C = false negatives, and D = false positives. Sensitivity = A/(A + C), Specificity = B/(B + D). Based on 100 cows (Healthy: 57, Mastitis: 43).
(DOCX)

S12 Table Sire-adjusted association analyses of candidate SNPs. Sire family distribution was evaluated to assess potential familial stratification within the study population. The two most represented sire families (12 and 7 offspring, respectively) included both healthy and mastitis cows, and no obvious clustering of mastitis cases within a major sire lineage was observed. These findings suggest that the associations identified in this study are unlikely to be explained solely by sire-family structure.
(XLSX)
