# Supplementary material for: Exploratory identification of candidate SNP markers associated with recurrent clinical mastitis in Holstein cattle
Source: PLoS One. 2026 Jul 30;21(7):e0355230. doi: 10.1371/journal.pone.0355230 (PMC13422837; doi:10.1371/journal.pone.0355230)
Supplement: S1 Fig — SNP1–SNP7 were included in the analysis. This figure shows the original uncropped and unadjusted gel images corresponding to the processed images presented in the Fig. 4. The electrophoresis image at the bottom left was excluded from the analysis. (PDF) [file pone.0355230.s001.pdf]

Miyata et al., S1 Figure .

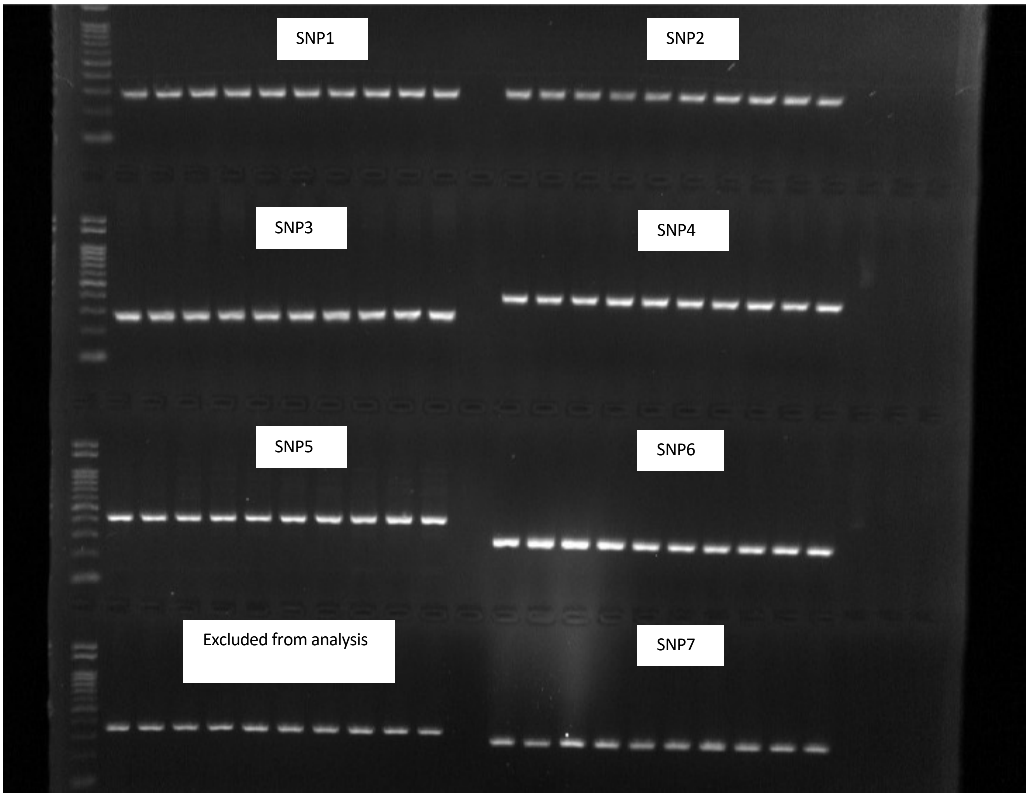

S1 Fig. The full-length gel image for seven SNPs.
SNP1–SNP7 were included in the analysis. The electrophoresis image at the bottom left was excluded from the analysis. This figure shows the original uncropped and unadjusted gel images corresponding to the processed images presented in the Fig. 4. The electrophoresis image at the bottom left was excluded from the analysis
