# Supplementary material for: Exploratory identification of candidate SNP markers associated with recurrent clinical mastitis in Holstein cattle
Source: PLoS One. 2026 Jul 30;21(7):e0355230. doi: 10.1371/journal.pone.0355230 (PMC13422837; doi:10.1371/journal.pone.0355230)
Supplement: S2 Fig — Sire family distribution was examined to evaluate potential familial stratification. The two most represented sire families (12 and 7 offspring, respectively) contained both healthy and mastitis animals, and no obvious clustering of mastitis cases within a single major sire lineage was observed. These observations suggest that the identified associations are unlikely to be explained solely by sire-family structure. (PDF) [file pone.0355230.s002.pdf]

Miyata et al., S2 Figure .

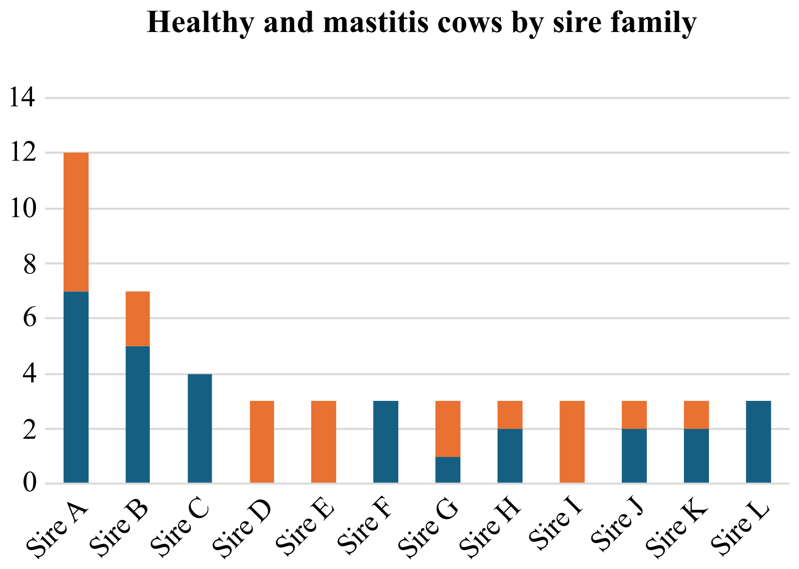

S2 Fig. Distribution of Healthy and Mastitis Cows by Sire Family. Sire family distribution was examined to evaluate potential familial stratification. The two most represented sire families (12 and 7 offspring, respectively) contained both healthy and mastitis animals, and no obvious clustering of mastitis cases within a single major sire lineage was observed. These observations suggest that the identified associations are unlikely to be explained solely by sire-family structure.
