# Supplementary material for: Exploratory identification of candidate SNP markers associated with recurrent clinical mastitis in Holstein cattle
Source: PLoS One. 2026 Jul 30;21(7):e0355230. doi: 10.1371/journal.pone.0355230 (PMC13422837; doi:10.1371/journal.pone.0355230)
Supplement: S4 Table — Required R² values to achieve 80% statistical power for a sample size of n = 50 under different significance levels (two-sided test). z₀.₈ = 0.8416. R² values are rounded to three significant figures. (DOCX) [file pone.0355230.s006.docx]

S4 Table. Required R2 for 80% power (n=50, two-sided).

Significance level (α, two-sided)  *z*_α/2_ (*z*_α/2_+ *z* _0.8_)^2^ ^†^ Required R^2^ for 80% power (N=50)

0.0001 3.890 22.75 0.455

0.001 3.291 18.77 0.375

0.00714 2.447 13.03 0.261

0.01 2.326 12.22 0.244

0.05 1.960 9.58 0.192

Required R² values to achieve 80% statistical power for a sample size of n = 50 under different significance levels (two-sided test). z₀.₈ = 0.8416. R² values are rounded to three significant figures.
