# Supplementary material for: Exploratory identification of candidate SNP markers associated with recurrent clinical mastitis in Holstein cattle
Source: PLoS One. 2026 Jul 30;21(7):e0355230. doi: 10.1371/journal.pone.0355230 (PMC13422837; doi:10.1371/journal.pone.0355230)
Supplement: S5 Table — Power analysis for SNP detection under varying effect sizes with n = 50 and α = 0.00714 (two-sided). Power was calculated for different assumed R² values. The non-centrality parameter (NCP) was calculated as √((N × R²)/(1 − R²)). (DOCX) [file pone.0355230.s007.docx]

S5 Table. Power analysis for SNP detection under varying effect sizes (n=50, α=0.00714).

Assumed R^2^ NCP^†^ Power^‡^

(Proportion of variance explained) (Non-Centrality Parameter) (α=0.00714, two-sided)

0.010 0.707 0.024

0.041 1.423 0.103

0.071 1.885 0.210

0.102 2.254 0.331

0.132 2.570 0.452

Power analysis for SNP detection under varying effect sizes with n = 50 and α = 0.00714 (two-sided). Power was calculated for different assumed R² values. The non-centrality parameter (NCP) was calculated as √((N × R²)/(1 − R²)).
