# Supplementary material for: Exploratory identification of candidate SNP markers associated with recurrent clinical mastitis in Holstein cattle
Source: PLoS One. 2026 Jul 30;21(7):e0355230. doi: 10.1371/journal.pone.0355230 (PMC13422837; doi:10.1371/journal.pone.0355230)
Supplement: S10 Table — Effects of SNP genotypes on milk composition parameters. Wild type represents reference homozygotes, whereas mutant includes both heterozygous and mutant homozygous genotypes. Asterisk indicates a statistically significant difference (P < 0.05). (DOCX) [file pone.0355230.s012.docx]

|  | Genotype | Mastitis (time) | Milk yield (kd/day) | Fat content (%) | Protein content (%) |
| --- | --- | --- | --- | --- | --- |
| SNP1 | Wild type (n=38) | 1.05±0.33 | 34.9±0.91 | 4.06±0.06 | 3.29±0.03 |
|  | Mutant (n=62) | 2.35±0.30* | 34.8±0.63 | 4.17±0.05 | 3.30±0.03 |
| SNP2 | Wild type (n=56) | 0.75±0.20 | 35.1±0.73 | 4.15±0.05 | 3.34±0.03 |
|  | Mutant (n=44) | 3.27±0.37* | 34.7±0.74 | 4.10±0.06 | 3.24±0.03* |
| SNP3 | Wild type (n=26) | 0.15±0.15 | 35.6±1.12 | 4.14±0.07 | 3.30±0.03 |
|  | Mutant (n=74) | 2.46±0.28* | 34.6±0.58 | 4.13±0.05 | 3.29±0.03 |
| SNP4 | Wild type (n=26) | 0.15±0.15 | 35.6±1.12 | 4.14±0.07 | 3.30±0.03 |
|  | Mutant (n=74) | 2.46±0.28* | 34.6±0.58 | 4.13±0.05 | 3.29±0.03 |
| SNP5 | Wild type (n=21) | 0 | 35.3±1.27 | 4.07±0.08 | 3.27±0.04 |
|  | Mutant (n=79) | 2.35±0.27* | 34.8±0.57 | 4.14±0.05 | 3.31±0.03 |
| SNP6 | Wild type (n=21) | 0 | 35.3±1.37 | 4.07±0.09 | 3.29±0.04 |
|  | Mutant (n=79) | 2.35±0.27* | 34.8±0.55 | 4.14±0.05 | 3.30±0.03 |
| SNP7 | Wild type (n=54) | 0.91±0.22 | 34.5±0.63 | 4.16±0.06 | 3.36±0.03 |
|  | Mutant (n=46) | 2.98±0.37* | 35.4±0.86 | 4.09±0.05 | 3.23±0.03* |

S10 Table. The effects of SNPs on key milk parameters-milk yield, fat content, and protein content.

Effects of SNP genotypes on milk composition parameters. Wild type represents reference homozygotes, whereas mutant includes both heterozygous and mutant homozygous genotypes. Asterisk indicates a statistically significant difference (P < 0.05).
