# Supplementary material for: Exploratory identification of candidate SNP markers associated with recurrent clinical mastitis in Holstein cattle
Source: PLoS One. 2026 Jul 30;21(7):e0355230. doi: 10.1371/journal.pone.0355230 (PMC13422837; doi:10.1371/journal.pone.0355230)
Supplement: S11 Table — Classification was based on a 2 × 2 contingency table, where A = true positives, B = true negatives, C = false negatives, and D = false positives. Sensitivity = A/(A + C), Specificity = B/(B + D). Based on 100 cows (Healthy: 57, Mastitis: 43). (DOCX) [file pone.0355230.s013.docx]

**S11 Table. Contingency table values for each SNP marker and SNP combinations.**

Classification was based on the following 2×2 contingency table:

|  | **Mastitis (n=43)** | **Healthy (n=57)** |
| --- | --- | --- |
| **Positive** (heterozygous or homozygous mutant) | A (True positive) | D (False positive) |
| **Negative** (reference homozygous) | C (False negative) | B (True negative) |

Sensitivity = A/(A+C), Specificity = B/(B+D)

| **SNP ID** | **A** | **B** | **C** | **D** | **Sensitivity** | **Specificity** |
| --- | --- | --- | --- | --- | --- | --- |
| SNP1 | 34 | 28 | 9 | 29 | 0.79 | 0.49 |
| SNP2 | 31 | 44 | 12 | 13 | 0.72 | 0.77 |
| SNP3 | 42 | 25 | 1 | 32 | 0.98 | 0.44 |
| SNP4 | 42 | 25 | 1 | 32 | 0.98 | 0.44 |
| SNP5 | 43 | 21 | 0 | 36 | 1.00 | 0.37 |
| SNP6 | 43 | 21 | 0 | 36 | 1.00 | 0.37 |
| SNP7 | 29 | 40 | 14 | 17 | 0.67 | 0.70 |
| SNP1+SNP7 | 26 | 50 | 17 | 7 | 0.60 | 0.88 |
| SNP2+SNP7 | 23 | 54 | 20 | 3 | 0.53 | 0.95 |
| SNP1+SNP2+SNP7 | 20 | 56 | 23 | 1 | 0.47 | 0.98 |
