# Supplementary material for: Exploratory identification of candidate SNP markers associated with recurrent clinical mastitis in Holstein cattle
Source: PLoS One. 2026 Jul 30;21(7):e0355230. doi: 10.1371/journal.pone.0355230 (PMC13422837; doi:10.1371/journal.pone.0355230)
Supplement: Attachment — Submitted filename: Review Report.docx [file pone.0355230.s015.docx]

**Reviewer # report:**

General comments:

The authors investigate potential SNP markers associated with recurrent clinical mastitis in Holstein cattle using whole-genome resequencing followed by validation. Mastitis remains a major economic and welfare concern in dairy farming, so identifying genetic markers for susceptibility is both timely and valuable. By focusing on recurrent cases within a single lactation, the study captures a more consistent and severe phenotype compared to research based solely on SCC measurements.

The study presents potentially interesting exploratory findings, particularly regarding X-linked SNPs and recurrent clinical mastitis, and while the authors acknowledge several limitations, some of their interpretations and conclusions stretch beyond what the data can truly support. The small sample size, limited statistical power for detecting individual SNPs, and lack of replication in external populations make it difficult to generalize or apply the findings widely. The manuscript would also benefit from thorough language editing to address typographical and formatting errors. Overall, it presents some intriguing early observations, especially regarding X-chromosome-linked variation, but requires major revisions to improve clarity, temper interpretations, and enhance overall presentation.

Addressing these points will boost the clarity and impact of the study. A thorough proofreading is needed to fix typos and maintain consistent formatting, making it easier to read.

Below are my major comments and minor suggestions for improvement:

1. The authors present a detailed post hoc power analysis showing very low power (under 20%) for individual SNP associations, but this limitation isn’t clearly highlighted in the discussion. While using polygenic risk score aggregation boosts statistical power, it doesn’t completely address concerns about false positives at the single-marker level.
2. Excluding subclinical mastitis cases creates a phenotype definition that differs from most mastitis GWAS studies. While this approach improves clinical specificity, it also makes comparisons with previous research more difficult and may exclude genetically significant cases. The reasoning behind this decision should be explained more clearly.
3. The chosen GWAS significance threshold (–log₁₀P > 5.0) is less stringent than the traditional Bonferroni correction based on the total number of SNPs tested. It would be useful to explain this choice more clearly and acknowledge the increased risk of false-positive associations.
4. One key finding is the clustering of significant SNPs on the X chromosome. However, the current analytical framework doesn’t specifically account for sex-specific inheritance, dosage compensation, or X-chromosome inactivation. These important biological and statistical factors deserve a deeper discussion.
5. Out of the 15 top SNPs chosen for validation, only 7 were successfully confirmed. The manuscript doesn’t mention why the other SNPs failed validation, such as potential primer design problems or sequencing quality issues. Including this detail would help in evaluating the study’s robustness.
6. The functional discussion relies largely on other studies including human studies (e.g., NXPE4) and doesn’t include cattle-specific functional or expression data. While this is fine for exploratory purposes, these interpretations should be clearly marked as speculative.
7. The validation relied on animals from the same herd, reducing both independence and broader population relevance. The absence of external replication should be more clearly acknowledged as a major limitation.

**Minor Comments**

**Introduction**

Line number 61-63: Sentence ending before citation [2] lacks punctuation. Add punctuation for sentence separation.

Line number 68-77: In this section, the author should emphasize in the introduction that genetic improvements for traits with low heritability and multifactorial nature can be enhanced through genomic or genetic selection.

Line number 77: Although it was emphasized that “Despite these advancements, reducing mastitis incidence through genomic selection remains challenging”, the author failed to explain why genomic selection remains challenging.

Line number 79-93: This section overemphasizes the limitations of SCC without clearly connecting them to the study’s genomic objective. The paragraph lacks a focused articulation of the specific knowledge gap that the present study aims to address, particularly with respect to recurrent clinical mastitis. While the paragraph argues that SCC is influenced by physiological factors (parity, age, stress, production), it **does not clearly link this limitation to the need for genomic markers**. The reader is left knowing that SCC is imperfect, but **not yet convinced why genetics, specifically SNP discovery, is the necessary solution.**

Lines number 86-91 mention recurrence, but the paragraph fails to emphasize why recurrence is biologically or genetically distinct from single episodes.

Line 94-96: The author mentioned that “In this study, mastitis -susceptible cows were defined as those that experienced three or more episodes of clinical mastitis (one episode includes onset, healing, and recurrence) within the same lactation period”. I would like to raise the point that if animals experience two episodes of clinical mastitis within the same lactation period, should they not be considered mastitis-susceptible? Could you clarify the rationale behind your definition of mastitis-susceptible animals?

**Materials and methods**

This manuscript presents serious problems in clearly describing and providing detailed models for GWAS analysis. Moreover, it lacks several critical methodological details and raises concerns that directly affect the reproducibility, rigor, and interpretability of the findings. The GWAS and variant filtering strategy is insufficiently transparent and deviates from common best practices. Key elements are missing or unclear, including: 1) the exact statistical model used for association testing (e.g., logistic regression, chi-square, mixed model), 2) whether population structure and relatedness were explicitly corrected beyond PCA visualization (e.g., inclusion of principal components or a kinship matrix in the model), and 3) justification for using a fixed –log₁₀P > 5.0 threshold rather than a genome-wide significance level derived from the actual number of independent tests. Addressing these issues is essential to substantiate the claim that the identified SNPs reflect true genetic susceptibility to recurrent clinical mastitis rather than herd-specific or management-driven effects.

Line number 117-122: “The TMR……. (2001) (S1 Table)”. This part needs to be paraphrased and re-written it to make it more understandable.

Line 136: “The top 10% of cows by parity were excluded”. What do you mean???

Line 139: “….. mastitis: n = 43) …. Which type of mastitis????

Line number 175-176: The author noted that “…. amplification success was confirmed via 2% agarose gel electrophoresis (S1 Fig). However, this sentence should be written more professionally, as agar gel electrophoresis is used to assess the quality of the amplified DNA.

Line 170: Title should be modified as the section states about validation of selected SNPs.

Line 183-185: The assumption that each SNP explains ~7% of phenotypic variance (R² ≈ 0.07) is optimistic and not well justified, particularly for a complex disease such as mastitis. No empirical or literature-based rationale is provided to support this effect size, which may lead to inflated power estimates, especially given the small sample size (n = 50).

Line number 190-193: The description of transforming odds ratios to liability-scale R², calculating non-centrality parameters (NCPs), and aggregating them into a polygenic score is overly technical and difficult to follow.

Line number 207: “PCA module in Python”. Incomplete sentence.

**Results**

Line number 221-224: Please re-write this sentence to make more readable.

Line number 232: Instead of writing fat and protein concentration, I suggest using fat and protein percentage, since these parameters are typically expressed as percentages.

Line number 256-260. Did the author examine the additive (a), dominant (d), and substitution (α) effects of these 15 highly significant SNPs? If not, I suggest doing so.

Line 264-265: “Herd traits, including key milk parameters and parity, are presented in S8 Table”.

I fail to understand the importance of this sentence in this section.

Line number 266-268: The authors reported that cows with homozygous or heterozygous genotypes exhibited a higher incidence of mastitis. Why are animals with these genotypes more prone to mastitis?

Line number 290-292: The diplotype analysis requires clearer explanation and more detailed justification, as it provides no meaningful insight in its current form. This should include how and why these SNPs were selected for diplotype analysis.

**Discussion**

Given the low power for individual SNP detection (<20%), the Discussion should frame these findings as hypothesis-generating rather than causal. The clinical relevance of the SNP panel is overstated, as its high specificity (98%) is offset by modest sensitivity (47%), limiting its value for selection or screening. The Discussion does not address the consequences of missing over half of susceptible cows, fails to compare this trade-off with existing breeding tools, and overlooks potential confounding between genetic susceptibility, infection persistence, and management-related recurrence.

Line number 312-313: This section fails to describe how the association analysis was performed and how it was selected for validation.

Line number 330-332: This discussion section refers to human studies, which may bias the interpretation when applied to bovines.

Line number 360-363: “Our results…..in dairy cattle.” This part needs to be supported and justified with relevant literature; otherwise, it remains a black box.

Line number 360-363: Is there any previous studies / reports with similar arguments???

**Conclusions and recommendations**

The authors attempted to highlight the limitations of this study; however, these limitations are not effectively integrated into the Discussion in relation to the significant SNPs. I strongly recommend strengthening the Discussion by explicitly incorporating the mentioned limitations.

**References**

The authors are solely responsible for following the journal’s writing guidelines for references.
