# Supplementary material for: Exploratory identification of candidate SNP markers associated with recurrent clinical mastitis in Holstein cattle
Source: PLoS One. 2026 Jul 30;21(7):e0355230. doi: 10.1371/journal.pone.0355230 (PMC13422837; doi:10.1371/journal.pone.0355230)
Supplement: Attachment — Submitted filename: Response Final .docx [file pone.0355230.s017.docx]

**Response to Editor:**

Comment#1

- Please ensure that your manuscript meets PLOS ONE's style requirements, including those for file naming. The PLOS ONE style templates can be found at https://journals.plos.org/plosone/s/file?id=wjVg/PLOSOne_formatting_sample_main_body.pdf and

https://journals.plos.org/plosone/s/file?id=ba62/PLOSOne_formatting_sample_title_authors_affiliations.pdf

[Response]

We thank the editor for this important comment. We have carefully revised the manuscript to ensure full compliance with PLOS ONE’s style requirements, including formatting and file naming in accordance with provided templates.

In addition to the revisions requested by the reviewers, we corrected an error in Table 4: the SNP combination previously listed as "SNP1+SNP2" has been corrected to "SNP1+SNP7" to accurately reflect the actual combination analyzed.

Comment#2

- We noticed you have some minor occurrence of overlapping text with the following previous publication(s), which needs to be addressed:

https://www.cell.com/heliyon/fulltext/S2405-8440(24)05820-1?_returnURL=https%3A%2F%2Flinkinghub.elsevier.com%2Fretrieve%2Fpii%2FS2405844024058201%3Fshowall%3Dtrue

In your revision ensure you cite all your sources (including your own works), and quote or rephrase any duplicated text outside the methods section. Further consideration is dependent on these concerns being addressed.

[Response]

We thank the editor for this important comment. We carefully reviewed the manuscript and identified overlapping text, particularly in the Material and Methods section in relation to our previous publication. Because the experimental design and animal cohort were similar to those used in our previous study, partial similarity in methodological description was unavoidable. However, the relevant sections have been thoroughly revised by rephrasing and restructuring the text to eliminate duplication. In addition, our previous study has now been appropriately cited where relevant. We confirm that all duplicated text has been addressed and that appropriate citations have been included. Please see the lines 110-113, 116-125, 128-139, 142-160.

Comment#3

- PLOS ONE now requires that authors provide the original uncropped and unadjusted images underlying all blot or gel results reported in a submission’s figures or Supporting Information files. This policy and the journal’s other requirements for blot/gel reporting and figure preparation are described in detail at https://journals.plos.org/plosone/s/figures#loc-blot-and-gel-reporting-requirements and https://journals.plos.org/plosone/s/figures#loc-preparing-figures-from-image-files. When you submit your revised manuscript, please ensure that your figures adhere fully to these guidelines and provide the original underlying images for all blot or gel data reported in your submission. See the following link for instructions on providing the original image data: https://journals.plos.org/plosone/s/figures#loc-original-images-for-blots-and-gels.

In your cover letter, please note whether your blot/gel image data are in Supporting Information or posted at a public data repository, provide the repository URL if relevant, and provide specific details as to which raw blot/gel images, if any, are not available. Email us at plosone@plos.org if you have any questions.

[Response]

We appreciate the editor’s helpful comment. We confirm that the original uncropped and unadjusted gel images corresponding to Fig.4 have been provided in the Supporting Information (S1). The corresponding figure legends have been revised to clearly indicate that these images represent the original data underlying the processed figures shown in the main manuscript.

Comment#4

- Thank you for stating the following financial disclosure:

“This study was supported by the Livestock Promotional Subsidies of the Japan Racing Association (JRA) (K.N.).”

Please state what role the funders took in the study. If the funders had no role, please state: "The funders had no role in study design, data collection and analysis, decision to publish, or preparation of the manuscript."

If this statement is not correct you must amend it as needed.

Please include this amended Role of Funder statement in your cover letter; we will change the online submission form on your behalf.

[Response]

We thank the editor for this important comment. The funding statement has been revised to clarify the role of the funder. The funders had no role in study design, data collection and analysis, decision to publish, or preparation of the manuscript.

Comment#5

- Please note that funding information should not appear in any section or other areas of your manuscript. We will only publish funding information present in the Funding Statement section of the online submission form. Please remove any funding-related text from the manuscript.

[Response]

We thank the editor for this important comment. All funding-related information has been removed from the manuscript text and is now reported only in the Funding Statement section of the online submission form, in accordance with PLOS ONE guidelines.

Comment#6

-Thank you for uploading your study's underlying data set. Unfortunately, the repository you have noted in your Data Availability statement does not qualify as an acceptable data repository according to PLOS's standards.

At this time, please upload the minimal data set necessary to replicate your study's findings to a stable, public repository (such as figshare or Dryad) and provide us with the relevant URLs, DOIs, or accession numbers that may be used to access these data. For a list of recommended repositories and additional information on PLOS standards for data deposition, please see https://journals.plos.org/plosone/s/recommended-repositories.

[Response]

We thank the editor for this comment. In response, we have deposited the minimal dataset necessary to replicate the findings of this study in figshare, which is listed among PLOS ONE's recommended repositories. The dataset is publicly available under the following DOI: https://doi.org/10.6084/m9.figshare.31829746. In addition, the variant call data remain available through the European Variation Archive (EVA) at EMBL-EBI under project accession number PRJEB88141. The Data Availability statement has been revised accordingly to include the repository name, accession information, and direct URL.

Comment#7

- In the online submission form you indicate that your data is not available for proprietary reasons and have provided a contact point for accessing this data. Please note that your current contact point is a co-author on this manuscript. According to our Data Policy, the contact point must not be an author on the manuscript and must be an institutional contact, ideally not an individual. Please revise your data statement to a non-author institutional point of contact, such as a data access or ethics committee, and send this to us via return email. Please also include contact information for the third party organization, and please include the full citation of where the data can be found.

[Response]

We thank the editor for this comment. As described in our response to Comment #6, the data underlying this study have now been deposited in a public repository figshare and are freely accessible without restriction. Because the data are now publicly available, a third-party institutional contact point for data access is no longer required. The Data Availability statement has been updated accordingly.

Comment#8

- If the reviewer comments include a recommendation to cite specific previously published works, please review and evaluate these publications to determine whether they are relevant and should be cited. There is no requirement to cite these works unless the editor has indicated otherwise.

[Response]

We appreciate the editor’s comment. We have reviewed the suggested references and determined that they are not directly relevant to the present study.

Comment#9

- Please review your reference list to ensure that it is complete and correct. If you have cited papers that have been retracted, please include the rationale for doing so in the manuscript text, or remove these references and replace them with relevant current references. Any changes to the reference list should be mentioned in the rebuttal letter that accompanies your revised manuscript. If you need to cite a retracted article, indicate the article’s retracted status in the References list and also include a citation and full reference for the retraction notice.

[Response]

We appreciate the editor’s comment. We have carefully reviewed the reference list to ensure that all citations are accurate and complete. No retracted articles were cited in this study. Any necessary corrections have been made in the revised manuscript.

**Response to reviewer1:**

General comments

Comment#1

- The authors investigate potential SNP markers associated with recurrent clinical mastitis in Holstein cattle using whole-genome resequencing followed by validation. Mastitis remains a major economic and welfare concern in dairy farming, so identifying genetic markers for susceptibility is both timely and valuable. By focusing on recurrent cases within a single lactation, the study captures a more consistent and severe phenotype compared to research based solely on SCC measurements.

[Response]

We appreciate the reviewer’s positive evaluation and recognition of the significance of our study.

Comment#2

- The study presents potentially interesting exploratory findings, particularly regarding X-linked SNPs and recurrent clinical mastitis, and while the authors acknowledge several limitations, some of their interpretations and conclusions stretch beyond what the data can truly support. The small sample size, limited statistical power for detecting individual SNPs, and lack of replication in external populations make it difficult to generalize or apply the findings widely. The manuscript would also benefit from thorough language editing to address typographical and formatting errors. Overall, it presents some intriguing early observations, especially regarding X-chromosome-linked variation, but requires major revisions to improve clarity, temper interpretations, and enhance overall presentation.

[Response]

Thank you so much for your thoughtful comments and review of the manuscript. We acknowledge the limitations of the study, including the relatively small sample size, limited statistical power, and lack of external validation. In response, we have revised the manuscript to provide a more cautious interpretation of the results and to clearly emphasize these limitations. In addition, we have carefully improved the clarity of the manuscript, including language editing and formatting corrections, to enhance overall readability and presentation.

Comment#3

- Addressing these points will boost the clarity and impact of the study. A thorough proofreading is needed to fix typos and maintain consistent formatting, making it easier to read.

[Response]

We appreciate the reviewer’s comment. We have carefully proofread the manuscript and corrected typographical errors and formatting inconsistencies to improve clarity and readability.

Major comments

Comment#1

- The authors present a detailed post hoc power analysis showing very low power (under 20%) for individual SNP associations, but this limitation isn’t clearly highlighted in the discussion. While using polygenic risk score aggregation boosts statistical power, it doesn’t completely address concerns about false positives at the single-marker level.

[Response]

Thank you so much for your important comment. We acknowledge that the statistical power for individual SNP associations was limited, as indicated by the post hoc power analysis. In response, we have revised the Discussion section to clearly highlight this limitation and to explicitly address the potential for false positives at the single-marker level. Please see the lines 495-510.

Comment#2

- Excluding subclinical mastitis cases creates a phenotype definition that differs from most mastitis GWAS studies. While this approach improves clinical specificity, it also makes comparisons with previous research more difficult and may exclude genetically significant cases. The reasoning behind this decision should be explained more clearly.

[Response]

We thank the reviewer’s important comment. We have clarified the rationale for excluding subclinical mastitis case in the manuscript. Specifically, we aimed to focus on clearly defined clinical phenotypes to improve phenotypic consistency. We have added a statement in the Materials and Methods section to explain this definition, and we have also added a statement in the Case Definition and Discussion to acknowledge that this approach may limit comparability with previous studies and may exclude genetically relevant cases. Please see the lines 134-137, 507-510.

Comment#3

- The chosen GWAS significance threshold (–log₁₀P > 5.0) is less stringent than the traditional Bonferroni correction based on the total number of SNPs tested. It would be useful to explain this choice more clearly and acknowledge the increased risk of false-positive associations.

[Response]

We appreciate the reviewer’s important comment. We have clarified the rationale for the chosen GWAS significance threshold in the Materials and Methods section. In addition, we have added a statement in the Discussion to acknowledge that this threshold is less stringent than a Bonferroni correction and may increase the risk of false positive associations. Please see the lines 503-510.

Comment#4

- One key finding is the clustering of significant SNPs on the X chromosome. However, the current analytical framework doesn’t specifically account for sex-specific inheritance, dosage compensation, or X-chromosome inactivation. These important biological and statistical factors deserve a deeper discussion.

[Response]

We appreciate the reviewer’s important comment. We acknowledge that the interpretation of X-linked SNP clustering is currently based on limited analytical considerations. We have revised the Discussion to clarify that these findings are preliminary and speculative, and to emphasize the need for further studies incorporating sex-specific inheritance, dosage compensation, and X-chromosome inactivation. Please see the lines 490-494.

Comment#5

-Out of the 15 top SNPs chosen for validation, only 7 were successfully confirmed. The manuscript doesn’t mention why the other SNPs failed validation, such as potential primer design problems or sequencing quality issues. Including this detail would help in evaluating the study’s robustness.

[Response]

we acknowledge that not all candidate SNPs were successfully validated. This was primarily due to technical limitations, including difficulties in primer design and sequencing quality issues. As reviewer requested, we have added a statement in the Discussion to clarify this point. Please see the lines 505-510.

Comment#6

- The functional discussion relies largely on other studies including human studies (e.g., NXPE4) and doesn’t include cattle-specific functional or expression data. While this is fine for exploratory purposes, these interpretations should be clearly marked as speculative.

[Response]

We appreciate the reviewer’s important comment. We acknowledge that the functional interpretation of candidate genes was primarily based on studies in other species. In response, we have revised the manuscript to clearly indicate that these interpretations are speculative and require further validation in cattle in Discussion. Please see the lines 440-441.

Comment#7

-The validation relied on animals from the same herd, reducing both independence and broader population relevance. The absence of external replication should be more clearly acknowledged as a major limitation.

[Response]

We appreciate the reviewer’s valuable comment. We acknowledge that the validation was conducted using animals from the same herd, which may limit independence and generalizability. We have added a statement in the Discussion to clearly highlight this as a limitation. Please see the lines 512-518.

Minor Comments

Introduction

Comment#1

- Line number 61-63: Sentence ending before citation [2] lacks punctuation. Add punctuation for sentence separation.

[Response]

As reviewer requested, we described punctuation. Please see the lines 51-54.

Comment#2

- Line number 68-77: In this section, the author should emphasize in the introduction that genetic improvements for traits with low heritability and multifactorial nature can be enhanced through genomic or genetic selection.

[Response]

We have revised the Introduction. Please see the lines 68-70.

Comment#3

- Line number 77: Although it was emphasized that “Despite these advancements, reducing mastitis incidence through genomic selection remains challenging”, the author failed to explain why genomic selection remains challenging.

[Response]

We appreciate the reviewer’s helpful comment. Please see the lines 68-70.

Comment#4

- Line number 79-93: This section overemphasizes the limitations of SCC without clearly connecting them to the study’s genomic objective. The paragraph lacks a focused articulation of the specific knowledge gap that the present study aims to address, particularly with respect to recurrent clinical mastitis. While the paragraph argues that SCC is influenced by physiological factors (parity, age, stress, production), it does not clearly link this limitation to the need for genomic markers. The reader is left knowing that SCC is imperfect, but not yet convinced why genetics, specifically SNP discovery, is the necessary solution.

[Response]

We appreciate the reviewer’s helpful comment.

We have revised the Introduction to more clearly link the limitations of SCC-based assessments to the need for genetic approaches, and to better highlight the knowledge gap addressed in this study. Please see the lines 85-87.

Comment#5

-Lines number 86-91 mention recurrence, but the paragraph fails to emphasize why recurrence is biologically or genetically distinct from single episodes.

[Response]

We thank the reviewer’s comment. We have revised the Introduction to clarify why recurrent mastitis represents a biologically and potentially genetically distinct phenotype compared to single episodes. Please see the lines 90-95.

Comment#6

- Line 94-96: The author mentioned that “In this study, mastitis -susceptible cows were defined as those that experienced three or more episodes of clinical mastitis (one episode includes onset, healing, and recurrence) within the same lactation period”. I would like to raise the point that if animals experience two episodes of clinical mastitis within the same lactation period, should they not be considered mastitis-susceptible? Could you clarify the rationale behind your definition of mastitis-susceptible animals?

[Response]

We appreciate the reviewer’s important comment. We have clarified the rationale for defining mastitis-susceptible cows as those with three or more episodes within a single lactation. A stricter threshold was applied to reduce phenotypic heterogeneity and to focus on individuals with a more consistent susceptibility to mastitis. We have added this explanation in the revised manuscript. Please see the lines 90-92.

Material method

Comment#7

- This manuscript presents serious problems in clearly describing and providing detailed models for GWAS analysis. Moreover, it lacks several critical methodological details and raises concerns that directly affect the reproducibility, rigor, and interpretability of the findings. The GWAS and variant filtering strategy is insufficiently transparent and deviates from common best practices. Key elements are missing or unclear, including: 1) the exact statistical model used for association testing (e.g., logistic regression, chi-square, mixed model), 2) whether population structure and relatedness were explicitly corrected beyond PCA visualization (e.g., inclusion of principal components or a kinship matrix in the model), and 3) justification for using a fixed –log₁₀P > 5.0 threshold rather than a genome-wide significance level derived from the actual number of independent tests. Addressing these issues is essential to substantiate the claim that the identified SNPs reflect true genetic susceptibility to recurrent clinical mastitis rather than herd-specific or management-driven effects.

[Response]

We thank the reviewer for these helpful comments regarding the clarity and transparency of the GWAS methodology. In response to these comments, we revised the Materials and Methods section to clarify the statistical model used for association testing, the evaluation of population structure, and the rationale for the significance threshold. We also clarified additional methodological details related to SNP quality control and added a statement in the Discussion acknowledging the limitations in statistical power associated with the relatively small sample size used for the genome-wide association analysis.

Specifically, the following revisions were made:

1) Statistical model used for association testing

We clarified that SNP associations were evaluated using a case–control association test (chi-square test) implemented in CLC Genomics Workbench. Please see the lines 200-205.

2) Population structure and relatedness correction

We added a description explaining that population structure was evaluated using principal component analysis (PCA) based on genome-wide SNP data. Because all animals originated from a single herd and no clear genetic stratification was observed in the PCA plot, no additional correction (e.g., inclusion of principal components or a kinship matrix) was applied. Please see the lines 243-248.

3) Justification of the significance threshold

We clarified that the threshold of –log₁₀(P) > 5 (P < 1 × 10⁻⁵) was used as an exploratory significance threshold for SNP discovery. Given the relatively small sample size and the exploratory nature of this study, this threshold was used to identify candidate loci for subsequent validation rather than to establish definitive genome-wide significance. Please see the lines 206-211.

Additional methodological clarification

To further improve transparency, we added a statement clarifying that only SNPs that passed stringent quality control filters during variant calling were retained for the association analysis. Please see the lines 206-211.

Discussion revision

We also added a statement in the Discussion acknowledging the limitation associated with the relatively small sample size used for the GWAS analysis and clarifying that the identified SNPs should be interpreted as candidate loci requiring validation in larger and independent populations. Please see the lines 495-502.

These revisions improve the transparency, reproducibility, and interpretability of the GWAS analytical framework used in this study.

Comment#8

- Line number 117-122: “The TMR……. (2001) (S1 Table)”. This part needs to be paraphrased and re-written it to make it more understandable.

[Response]

As reviewer requested, we have revised the relevant sentences to improve clarity and readability. Please see the lines 121-125.

Comment#9

Line 136: “The top 10% of cows by parity were excluded”. What do you mean???

[Response]

As reviewer requested, we have revised the relevant sentences to improve clarity and readability. Please see the lines 147-149.

Comment#10

- Line number 175-176: The author noted that “…. amplification success was confirmed via 2% agarose gel electrophoresis (S1 Fig). However, this sentence should be written more professionally, as agar gel electrophoresis is used to assess the quality of the amplified DNA.

[Response]

We thank the reviewer’s helpful comment. We have revised the sentence to more accurately describe the purpose of agarose gel electrophoresis in assessing the quality and size of the amplified DNA. Please see the lines 193-196

Comment#11

- Line 170: Title should be modified as the section states about validation of selected SNPs.

[Response]

We appreciate the reviewer’s helpful comment. We have revised the section title to more accurately reflect the content, which focuses on the validation of candidate SNP markers. Please see the line 190.

Comment#12

- Line 183-185: The assumption that each SNP explains ~7% of phenotypic variance (R² ≈ 0.07) is optimistic and not well justified, particularly for a complex disease such as mastitis. No empirical or literature-based rationale is provided to support this effect size, which may lead to inflated power estimates, especially given the small sample size (n = 50).

[Response]

We thank the reviewer for this comment. We agree that the assumed effect size (R² ≈ 0.07) may appear optimistic for a complex trait such as mastitis. In the revised manuscript, we clarified that this value was used only as an illustrative assumption to estimate statistical power under the present sample size rather than as an empirically established effect size. We also moderated the interpretation of the power analysis accordingly. Revised in Materials and Methods (Lines 213-221).

Comment#13

- Line number 190-193: The description of transforming odds ratios to liability-scale R², calculating non-centrality parameters (NCPs), and aggregating them into a polygenic score is overly technical and difficult to follow.

[Response]

We thank the reviewer for this suggestion. To improve clarity and readability, we simplified the description of the statistical power calculations by removing overly technical details and summarizing the analytical approach in a more concise manner. Revised in Materials and Methods (Lines 213-221).

Comment#14

-Line number 207: “PCA module in Python”. Incomplete sentence.

[Response]

We thank the reviewer for identifying this issue. The sentence describing the PCA analysis has been corrected for clarity. Revised in Materials and Methods (Lines 243-248).

Comment#15

- Line number 221-224: Please re-write this sentence to make more readable.

[Response]

A reviewer requested, we have revised the sentence to improve clarity and readability.

Please see the lines 262-266.

Comment#16

- Line number 232: Instead of writing fat and protein concentration, I suggest using fat and protein percentage, since these parameters are typically expressed as percentages.

[Response]

As reviewer requested, we revised concentration to percent. Please see the line 274.

Comment#17

-Line number 256-260. Did the author examine the additive (a), dominant (d), and substitution (α) effects of these 15 highly significant SNPs? If not, I suggest doing so.

[Response]

We appreciate the reviewer’s valuable suggestion. The primary aim of this study was to identify SNPs associated with mastitis susceptibility using GWAS. While estimation of additive, dominant, and substitution effects is important in the context of animal breeding, it was beyond the scope of the present study. This will be considered in future work.

Comment#18

- Line 264-265: “Herd traits, including key milk parameters and parity, are presented in S8 Table”.

I fail to understand the importance of this sentence in this section.

[Response]

We appreciate the reviewer’s comment. We have revised the sentence to clarify that S8 Table provides background characteristics of the validation population. Please see the lines 347-348.

Comment#19

- Line number 266-268: The authors reported that cows with homozygous or heterozygous genotypes exhibited a higher incidence of mastitis. Why are animals with these genotypes more prone to mastitis?

[Response]

We appreciate the reviewer’s important comment. We acknowledge that the biological mechanisms underlying these associations are not fully understood. We have added a statement in the Discussion to clarify that these findings may reflect underlying genetic susceptibility, but that further functional studies are required.

Please see the lines 426-430.

Comment#20

- Line number 290-292: The diplotype analysis requires clearer explanation and more detailed justification, as it provides no meaningful insight in its current form. This should include how and why these SNPs were selected for diplotype analysis.

[Response]

We thank the reviewer’s important comment. We have revised the manuscript to clarify the rationale for SNP selection in the combination analysis and to better explain the purpose of this analysis. Please see the lines 378-382.

Discussion

Comment#21

- Given the low power for individual SNP detection (<20%), the Discussion should frame these findings as hypothesis-generating rather than causal. The clinical relevance of the SNP panel is overstated, as its high specificity (98%) is offset by modest sensitivity (47%), limiting its value for selection or screening. The Discussion does not address the consequences of missing over half of susceptible cows, fails to compare this trade-off with existing breeding tools, and overlooks potential confounding between genetic susceptibility, infection persistence, and management-related recurrence.

[Response]

We appreciate the reviewer’s important comment. We have revised the Discussion to more clearly present the findings as hypothesis-generating, to address the limitations related to sensitivity and clinical applicability, and to discuss potential cofounding factors such as infection persistence and management condition. Please see the lines 408-409, 418-421, 440-441,464-466.

Comment#22

-Line number 312-313: This section fails to describe how the association analysis was performed and how it was selected for validation.

[Response]

We thank the reviewer’s comment. We have revised the manuscript to clarify how the SNPs were identified and selected for validation based on GWAS analysis and statistical criteria. Please see the lines 406-409.

Comment#23

- Line number 360-363: “Our results…..in dairy cattle.” This part needs to be supported and justified with relevant literature; otherwise, it remains a black box.

[Response]

We thank the reviewer’s comment. Please see the Major commnet#6.

Comment#24

- Line number 360-363: “Our results…..in dairy cattle.” This part needs to be supported and justified with relevant literature; otherwise, it remains a black box.

[Response]

We appreciate the reviewer’s important comment. We have revised the sentence to avoid overinterpretation and to more appropriately reflect the level

Of evidence, while supporting the discussion with relevant literature as described in the following section (lines 480-481). Please see the lines 471-473.

Comment#25

- Line number 360-363: Is there any previous studies / reports with similar arguments???

[Response]

Please see the Comment#23.

Conclusion and recommendations

Comment#26

- The authors attempted to highlight the limitations of this study; however, these limitations are not effectively integrated into the Discussion in relation to the significant SNPs. I strongly recommend strengthening the Discussion by explicitly incorporating the mentioned limitations.

[Response]

We appreciate the reviewer’s valuable comment. We have revised the Discussion to more effectively integrated the study limitations into the interpretation of the identified SNPs. In particular, we have clarified the implications of limited statistical power, moderate sensitivity, validation within a single herd, and potential cofounding factors in relation to the reported findings.

Comment#27

-The authors are solely responsible for following the journal’s writing guidelines for references.

[Response]

We thank the reviewer’s comment. We have carefully reviewed the journal’s reference formatting guidelines and revised the reference list accordingly.

**Response to reviewer2:**

Comment#1

- This paper describes a GWAS analysis to identify mastitis markers that can aid herd management and selection. They start with providing a definition of mastitis-susceptibility. They utilized 50 genome sequences to filter SNP candidates, and used another set of 100 animals to validate the association. They propose 7 SNP markers to be combined in sets of 2-3 markers to screen with confidence for animals with genetic predisposition to mastitis. This study deserves to be published and can benefit the dairy industry.

[Response]

We appreciate the reviewer’s positive evaluation of our study and their recognition of its potential contribution to dairy herd management and selection.

Minor comments

Conclusion

Comment#1

- Line 391 - 395

I suggest to transfer the 'In conclusion' phrase to the second sentence. To read: "In conclusion, this study suggest a contribution of X-linked genetic variation to mastitis

susceptibility and provide preliminary markers that may aid in the development of

genomic selection panels."

[Response]

We thank the reviewer’s helpful suggestion. We have revised the sentence by repositing “In conclusion” to improve clarity and flow. Please see the lines 519-523.

Result

Comment#2

- All figure titles (Fig 1 -6) could be improved by adding short description of the results. It is a general rule that the tables and figures be able to stand-alone (meaning, without need for reading the body of the manuscript). Thus, all details are included especially a short description of the result. Here are examples:

In Figure 2. Principal component analysis (PCA) of SNP data showed no significant differences in SNP distribution between healthy and mastitis group.

In Figure 3. Genome-wide association analysis identified 15 highly associated SNPs for further validation.

In Figure 1 - please indicate how many animals were sequenced? Also indicate this data in Materials and Methods.

[Response]

We thank the reviewer’s helpful comment. We have revised all figure legends to include brief descriptions of the main results, ensuring that each figure can be understood independently of the main text. We have also clarified the number of animals used in Figure 1 and in the Materials and Methods section. Please see the line 163 and lines 298-304, 305-310, 311-317, 318-322, 336-342, 358-361.

Comment#3

- Table titles also need to be improved for better readability.

Table 3. Include number of animals in the footnote. "Based from 100 cows."

Table 2. Seven candidate SNP markers associated with mastitis susceptibility were identified by genome-wide association analysis. Also, please include a row in Table 2 showing allele frequency distribution from the sequencing result of 100 animals.

[Response]

We thank the reviewer’s helpful comments. We have revised the table titles. We have added the number of animals in the footnote of Table 3. In Table 2, we have clarified the description and included allele frequency data derived from the sequencing results of 100 animals. Please see the Table 2 (327-330) and Table 3 (367-368).

Discussion

Comment#4

- Line 362 - it is best to keep discussion within the scope of the study, thus, specify mastitis. to read: "a crucial role in determining mastitis immune response"

[Response]

We appreciate the reviewer’s important comment. We have revised the sentence to avoid overinterpretation and to more appropriately reflect the level

Of evidence, while supporting the discussion with relevant literature as described in the following section (lines 480-481). Please see the lines 471-473.

Comment#5

- Line 365: indicate the meaning of lower p-values, to read: allowing us to identify variants with lower p-values (highly significant).

[Response]

We appreciate the reviewer’s helpful comment. We have revised the sentence to clarify the meaning of lower p-values by indicating their higher statistical significance. Please see the line 464.

Comment#6

- Line 352: same comment: "high p-values (low significance)"

[Response]

We have revised the sentence in the same manner as described in Comment #5. Please see the line 459.

Comment#7

- Line 325: I suggest this revision: "for the early identification of cows with genetic predisposition to recurrent mastitis."

[Response]

As reviewer suggestion, we have revised sentence. Please see the lines 424-425.

Comment#8

-Line 318: Did you mean heterozygote and homozygote mutant genotype? I suggest this revision: "cows carrying a mutant allele were considered"

[Response]

We thank the reviewer’s comment. We have revised sentences. Please see the line 416.

Comment#9

- Line 307: I suggest this revision: "false positives in traditional susceptibility screening".

[Response]

As reviewer’s suggestion, we have revised sentences. Please see the line 401.

MATERIALS AND METHODS

Comment#10

- For those who do not understand sensitivity and specificity scores, it would be nice to add a Supplementary table showing the 2x2 contingency table or confusion matrix how you arrive to those values, for each SNP. Also add a 1 line discussion on the relevance of the kappa coefficient.

[Response]

We appreciate the reviewer's helpful comment. In response, we have added a new supplementary table (S10 Table) presenting the 2×2 contingency table values (true positives, true negatives, false negatives, and false positives) for each individual SNP and SNP combination. This allows readers to verify how sensitivity and specificity values were derived. In addition, we have clarified the description of sensitivity and specificity in the Materials and Methods section and added a brief explanation of the relevance of Cohen's kappa coefficient in the Discussion (lines 411-413). Please see the Materials and Methods, Results (Table 3), and S10 Table.

Comment#11

- Please add a line or related articles justifying that a significance threshold of p-value< 1x10^-6 is sufficient for whole genome-based SNPs? It is definitely higher than Kurz et al 2019 which set it at 1x10^-4 but which also used a SNP Chip BovineHD, but industry standard is supposedly set at 1x10^-8. See ref: <https://doi.org/10.1093/genetics/iyaf056>

[Response]

We thank the reviewer’s important comment. In the present study, we applied a suggestive threshold of p<1×10^-6^ to identify candidate SNPs associated with mastitis susceptibility. Similar thresholds have been used in exploratory GWAS studies with relatively small sample sizes. We have clarified this rationale and added supporting references in the revised manuscript. Please see the lines 209-211.

Comment#12

- Line 171 ... "were validated by PCR and Sanger sequencing."

[Response]

As reviewer’s suggestion, we have revised sentence. Please see the lines 191-192.

Comment#13

- Bos taurus and ad libitum should be italicized.

[Response]

We thank the reviewer’s thoughtful comment. We have revised the terminology. Please see the line 120, 178.

ABSTRACT

Comment#14

-Line 41 should include mastitis, to read "mastitis immune response..". Best to avoid over generalization.

[Response]

We thank the reviewer’s important comment. We have revised sentence. Please see the line 40.
