# Supplementary material for: Exploratory identification of candidate SNP markers associated with recurrent clinical mastitis in Holstein cattle
Source: PLoS One. 2026 Jul 30;21(7):e0355230. doi: 10.1371/journal.pone.0355230 (PMC13422837; doi:10.1371/journal.pone.0355230)
Supplement: Attachment — Submitted filename: Response .docx [file pone.0355230.s018.docx]

Editor request

If data are available, it would also be interesting to more precisely define the mastitis cases by indicating which pathogen was responsible for mastitis cases recorded.

Reply:

We thank the editor for this suggestion. Bacteriological culture data were available for the 25 mastitis-susceptible cows in the discovery cohort. The most frequently isolated pathogens were coagulase-negative staphylococci (CNS; 15/25 cows), *Escherichia coli* (12/25), *Streptococcus* spp. (12/25), and *Klebsiella* spp. (11/25). Notably, 21 of 25 cows experienced mastitis caused by multiple different pathogen species across episodes, suggesting that the recurrent mastitis phenotype in these animals is more likely attributable to host-related susceptibility factors than to persistent infection by a single pathogen. These data have been added to the Results section and a detailed summary is provided in new S7 Table. Please see lines 293-299.

To Reviewer3

My main concern remains the GWAS analytical framework. Although the authors clarified that CLC Genomics Workbench was used for the GWAS, the issue is not the use of the software itself, but whether the specific case–control chi-square association test is sufficient for this dataset. Recurrent clinical mastitis is a complex and multifactorial disease trait influenced by genetic, physiological, environmental, and management-related factors. Given the small sample size, same-herd design, and potential cryptic relatedness among animals, a simple chi-square test may not adequately control false-positive associations.
If the authors wish to retain this analytical framework, they should provide stronger justification and, ideally, cite studies where CLC Genomics Workbench was specifically used for GWAS association testing (livestock research), not only for read quality control, mapping, or variant calling. Alternatively, the authors should consider reanalyzing the data using a mixed-model GWAS framework that can account for relatedness, population structure, and relevant covariates. For example, depending on the available pedigree, genotype, and phenotype data, software commonly used in animal breeding and genomic evaluation, such as the BLUPF90 family of programs, could be considered. Since recurrent mastitis may be treated either as a binary disease phenotype or as a count trait based on the number of episodes, the statistical model should be selected accordingly.

Reply:

We acknowledge the reviewer's concern. To address this, we performed GLMM analyses including sire as a random effect. All seven candidate SNPs remained significantly associated with mastitis status (S12 Table). Poisson mixed-effects models for mastitis episode count also confirmed significant associations for all SNPs (S12 Table). The estimated sire variance was negligible, supporting the robustness of the identified associations. Complete pedigree information for a full mixed-model GWAS (e.g., BLUPF90) was not available, which is acknowledged as a limitation. Please see the Materials and Methods and Results sections. Please see lines 260-268, 412-420.

The authors state that no additional correction for population structure was applied because all animals originated from a single herd and no clear stratification was observed in the PCA plot. However, PCA visualization alone is not sufficient to exclude cryptic relatedness or subtle family structure, particularly in a small Holstein dataset. Animals within the same herd may still be related through common sires, dams, or breeding lines. A genomic relationship or kinship matrix can be estimated from genome-wide SNP data and incorporated into a mixed-model GWAS to account for pairwise relatedness among animals. The authors should either apply such correction or explicitly acknowledge the lack of kinship correction as a major limitation.

Reply:

We agree that PCA alone cannot exclude cryptic relatedness. To address this, we obtained sire information for all 100 cows and confirmed that the two largest sire families contained both healthy and mastitis cows, with no obvious clustering of mastitis cases (S2 Fig). Furthermore, the GLMM analyses described above (Comment 1) included sire as a random effect, and the estimated sire variance was negligible. Although a full genomic relationship matrix could not be constructed from the available data, the lack of kinship correction in the primary GWAS has been acknowledged as a limitation in the Discussion. Please see lines 535-540.

The statement regarding Bonferroni correction and the –log₁₀(P) threshold should also be clarified. In the Materials and Methods section, the statement in lines 186–189 should be moved to the Statistical Analysis section, as this pertains to GWAS association testing rather than variant calling or annotation. More importantly, the threshold of –log₁₀(P) > 5.0 does not correspond to a Bonferroni-corrected genome-wide significance threshold for the 536,184 SNPs retained after quality control. If the authors used –log₁₀(P) > 5.0 as an exploratory threshold to prioritize candidate SNPs, this should be stated explicitly. I suggest removing the claim that Bonferroni correction was applied unless the authors provide the actual Bonferroni-corrected threshold and explain how it was used in the analysis.

Reply:

We agree with the reviewer. The –log₁₀(P) > 5.0 threshold was used as an exploratory threshold to prioritize candidate SNPs, not as a Bonferroni-corrected genome-wide significance level. We have removed the claim of Bonferroni correction, clarified the exploratory nature of the threshold, and moved the relevant statement from the Sequence Data Analysis section to the Statistical Analysis section. Please see lines 254-259.

The Q-Q plot should also be interpreted more cautiously. Several livestock GWAS studies routinely report the genomic inflation factor, λ, together with Q-Q plots to evaluate whether association statistics are inflated by population structure, relatedness, or model misspecification. I suggest that the authors report λ for their GWAS results. This is particularly important because the Q-Q plot appears to show some upward deviation, and the current analysis did not include a kinship matrix or principal components as covariates. Reporting λ would help readers assess whether the chi-square association test was adequately calibrated.

Reply:

We thank the reviewer for this suggestion. The present study was designed as an exploratory WGS-based candidate variant identification study rather than a conventional genome-wide association study. Therefore, λ was not calculated in the original analysis pipeline. In addition, the archived association results contained only variants that passed significance filtering, and the complete genome-wide P-value distribution was not retained for retrospective analysis. To directly address the reviewer's concern regarding potential confounding by relatedness, we performed pedigree-based mixed-effects analyses. GLMMs with sire as a random effect confirmed that all seven candidate SNPs remained significantly associated with mastitis status, and Poisson mixed-effects models yielded consistent results for mastitis episode counts (S12 Table). The estimated sire variance was negligible, indicating that the observed associations are robust to familial relatedness. We have acknowledged the inability to report λ as a limitation in the Discussion. Please see lines 540-543.

The wording regarding SNP effects should be revised. In line 262, the statement that “each individual SNP explained approximately 7% of the phenotypic variance” may be misleading because it implies that this value was empirically estimated from the data. However, based on the Methods section, the 7% value appears to have been used as an assumed or illustrative effect size for the statistical power calculation. The authors should revise this wording to clearly indicate that R² ≈ 0.07 was an assumption used for power analysis, not an observed estimate of phenotypic variance explained by each SNP. Alternatively, this statement should be removed if the authors cannot provide an empirical basis for the 7% estimate.

Reply:

We agree that the original wording was misleading. We have revised the statement to clarify that R² ≈ 0.07 was an assumed effect size used for statistical power estimation, not an empirically observed value. Please see the Results section. Please see lines 272-276.

The validation analysis also requires clarification. The authors should clearly state whether the phenotype analyzed in the validation step was mastitis status or the number of mastitis episodes. If the outcome was mastitis incidence/status, logistic regression, chi-square test, or Fisher’s exact test would be more appropriate. If the outcome was the number of mastitis episodes, as shown in the figure, then the trait is count data; therefore, Poisson regression or negative binomial regression would be more suitable than Kruskal–Wallis, particularly if overdispersion is present. The authors should justify the statistical test used and report effect estimates with confidence intervals and adjusted P-values where possible.

Reply:

We thank the reviewer for this clarification. In the original validation analysis, the Kruskal–Wallis test was used as a non-parametric approach to compare mastitis episode counts across genotype groups (Fig. 6). To address the reviewer's concern, we performed additional analyses using GLMM with mastitis status as a binary outcome and a Poisson mixed-effects model with mastitis episode count as a count outcome, both including sire as a random effect (S12 Table). These analyses confirmed significant associations for all seven candidate SNPs. The Methods sections have been updated accordingly. Please see lines 243-247.

I also suggest replacing the term “significant SNPs” with more cautious wording such as “candidate SNPs,” “putative SNPs,” or “putative mastitis-associated SNPs.” Given the exploratory threshold, limited sample size, same-herd validation, and lack of external independent replication, the current wording may overstate the strength of evidence. Similarly, terms such as “validated markers,” “predictive panel,” and “marker-assisted selection” should be softened unless supported by stronger statistical evidence and external validation.
Reply:

We agree with the reviewer. Throughout the manuscript, we have replaced "significant SNPs" with "candidate SNPs" or "putative mastitis-associated SNPs," softened "validated markers" to "candidate markers evaluated in the validation cohort," replaced "predictive panel" with "candidate marker panel," and used "marker-assisted selection" only in the context of future prospects. These changes have been applied consistently throughout the Abstract, Results, and Discussion.
