# Supplementary material for: Exploratory identification of candidate SNP markers associated with recurrent clinical mastitis in Holstein cattle
Source: PLoS One. 2026 Jul 30;21(7):e0355230. doi: 10.1371/journal.pone.0355230 (PMC13422837; doi:10.1371/journal.pone.0355230)
Supplement: Peer review history

# Peer review: Decision Letter 0

**DOI:** 10.1371/journal.pone.0355230.r001

**Authors:** Amod Kumar

11 Feb 2026

PONE-D-25-49503

Exploratory identification of candidate SNP markers associated with recurrent clinical mastitis in Holstein cattle

PLOS One

Dear Dr. Nagaoka,

Thank you for submitting your manuscript to PLOS ONE. After careful consideration, we feel that it has merit but does not fully meet PLOS ONE’s publication criteria as it currently stands. Therefore, we invite you to submit a revised version of the manuscript that addresses the points raised during the review process.

Please submit your revised manuscript by Mar 28 2026 11:59PM. If you will need more time than this to complete your revisions, please reply to this message or contact the journal office at plosone@plos.org. When you’re ready to submit your revision, log on to https://www.editorialmanager.com/pone/ and select the 'Submissions Needing Revision' folder to locate your manuscript file.

Please include the following items when submitting your revised manuscript:

- A letter that responds to each point raised by the academic editor and reviewer(s). You should upload this letter as a separate file labeled 'Response to Reviewers'.
- A marked-up copy of your manuscript that highlights changes made to the original version. You should upload this as a separate file labeled 'Revised Manuscript with Track Changes'.
- An unmarked version of your revised paper without tracked changes. You should upload this as a separate file labeled 'Manuscript'.

-->If you would like to make changes to your financial disclosure, please include your updated statement in your cover letter. Guidelines for resubmitting your figure files are available below the reviewer comments at the end of this letter.

If applicable, we recommend that you deposit your laboratory protocols in protocols.io to enhance the reproducibility of your results. Protocols.io assigns your protocol its own identifier (DOI) so that it can be cited independently in the future. For instructions see: https://journals.plos.org/plosone/s/submission-guidelines#loc-laboratory-protocols. Additionally, PLOS ONE offers an option for publishing peer-reviewed Lab Protocol articles, which describe protocols hosted on protocols.io. Read more information on sharing protocols at https://plos.org/protocols?utm_medium=editorial-email&utm_source=authorletters&utm_campaign=protocols.

We look forward to receiving your revised manuscript.

Kind regards,

Amod Kumar, Ph.D

Academic Editor

PLOS One

Journal Requirements:

When submitting your revision, we need you to address these additional requirements.

1.Please ensure that your manuscript meets PLOS ONE's style requirements, including those for file naming. The PLOS ONE style templates can be found at

https://journals.plos.org/plosone/s/file?id=wjVg/PLOSOne_formatting_sample_main_body.pdf and

https://journals.plos.org/plosone/s/file?id=ba62/PLOSOne_formatting_sample_title_authors_affiliations.pdf

2. We noticed you have some minor occurrence of overlapping text with the following previous publication(s), which needs to be addressed:

https://www.cell.com/heliyon/fulltext/S2405-8440(24)05820-1?_returnURL=https%3A%2F%2Flinkinghub.elsevier.com%2Fretrieve%2Fpii%2FS2405844024058201%3Fshowall%3Dtrue

In your revision ensure you cite all your sources (including your own works), and quote or rephrase any duplicated text outside the methods section. Further consideration is dependent on these concerns being addressed.

3. PLOS ONE now requires that authors provide the original uncropped and unadjusted images underlying all blot or gel results reported in a submission’s figures or Supporting Information files. This policy and the journal’s other requirements for blot/gel reporting and figure preparation are described in detail at https://journals.plos.org/plosone/s/figures#loc-blot-and-gel-reporting-requirements and https://journals.plos.org/plosone/s/figures#loc-preparing-figures-from-image-files. When you submit your revised manuscript, please ensure that your figures adhere fully to these guidelines and provide the original underlying images for all blot or gel data reported in your submission. See the following link for instructions on providing the original image data: https://journals.plos.org/plosone/s/figures#loc-original-images-for-blots-and-gels.

In your cover letter, please note whether your blot/gel image data are in Supporting Information or posted at a public data repository, provide the repository URL if relevant, and provide specific details as to which raw blot/gel images, if any, are not available. Email us at plosone@plos.org if you have any questions.

4. Thank you for stating the following financial disclosure:

“This study was supported by the Livestock Promotional Subsidies of the Japan Racing Association (JRA) (K.N.).”

Please state what role the funders took in the study.  If the funders had no role, please state: "The funders had no role in study design, data collection and analysis, decision to publish, or preparation of the manuscript."

If this statement is not correct you must amend it as needed.

Please include this amended Role of Funder statement in your cover letter; we will change the online submission form on your behalf.

5. Please note that funding information should not appear in any section or other areas of your manuscript. We will only publish funding information present in the Funding Statement section of the online submission form. Please remove any funding-related text from the manuscript.

6. Thank you for uploading your study's underlying data set. Unfortunately, the repository you have noted in your Data Availability statement does not qualify as an acceptable data repository according to PLOS's standards.

At this time, please upload the minimal data set necessary to replicate your study's findings to a stable, public repository (such as figshare or Dryad) and provide us with the relevant URLs, DOIs, or accession numbers that may be used to access these data. For a list of recommended repositories and additional information on PLOS standards for data deposition, please see https://journals.plos.org/plosone/s/recommended-repositories.

7. In the online submission form you indicate that your data is not available for proprietary reasons and have provided a contact point for accessing this data. Please note that your current contact point is a co-author on this manuscript. According to our Data Policy, the contact point must not be an author on the manuscript and must be an institutional contact, ideally not an individual. Please revise your data statement to a non-author institutional point of contact, such as a data access or ethics committee, and send this to us via return email. Please also include contact information for the third party organization, and please include the full citation of where the data can be found.

8. If the reviewer comments include a recommendation to cite specific previously published works, please review and evaluate these publications to determine whether they are relevant and should be cited. There is no requirement to cite these works unless the editor has indicated otherwise.

9. Please review your reference list to ensure that it is complete and correct. If you have cited papers that have been retracted, please include the rationale for doing so in the manuscript text, or remove these references and replace them with relevant current references. Any changes to the reference list should be mentioned in the rebuttal letter that accompanies your revised manuscript. If you need to cite a retracted article, indicate the article’s retracted status in the References list and also include a citation and full reference for the retraction notice.

[Note: HTML markup is below. Please do not edit.]

Reviewer's Responses to Questions

**Comments to the Author**

1. Is the manuscript technically sound, and do the data support the conclusions?

The manuscript must describe a technically sound piece of scientific research with data that supports the conclusions. Experiments must have been conducted rigorously, with appropriate controls, replication, and sample sizes. The conclusions must be drawn appropriately based on the data presented.

Reviewer #1: Partly

Reviewer #2: Yes

**********

2. Has the statistical analysis been performed appropriately and rigorously?

Reviewer #1: No

Reviewer #2: I Don't Know

**********

3. Have the authors made all data underlying the findings in their manuscript fully available?

The PLOS Data policy requires authors to make all data underlying the findings described in their manuscript fully available without restriction, with rare exception (please refer to the Data Availability Statement in the manuscript PDF file). The data should be provided as part of the manuscript or its supporting information, or deposited to a public repository. For example, in addition to summary statistics, the data points behind means, medians and variance measures should be available. If there are restrictions on publicly sharing data—e.g. participant privacy or use of data from a third party—those must be specified.

Reviewer #1: No

Reviewer #2: Yes

**********

4. Is the manuscript presented in an intelligible fashion and written in standard English?

PLOS ONE does not copyedit accepted manuscripts, so the language in submitted articles must be clear, correct, and unambiguous. Any typographical or grammatical errors should be corrected at revision, so please note any specific errors here.

Reviewer #1: Yes

Reviewer #2: Yes

**********

5. Review Comments to the Author

Please use the space provided to explain your answers to the questions above. You may also include additional comments for the author, including concerns about dual publication, research ethics, or publication ethics. (Please upload your review as an attachment if it exceeds 20,000 characters)

Reviewer #1: Reviewer # report:

General comments:

The authors investigate potential SNP markers associated with recurrent clinical mastitis in Holstein cattle using whole-genome resequencing followed by validation. Mastitis remains a major economic and welfare concern in dairy farming, so identifying genetic markers for susceptibility is both timely and valuable. By focusing on recurrent cases within a single lactation, the study captures a more consistent and severe phenotype compared to research based solely on SCC measurements.

The study presents potentially interesting exploratory findings, particularly regarding X-linked SNPs and recurrent clinical mastitis, and while the authors acknowledge several limitations, some of their interpretations and conclusions stretch beyond what the data can truly support. The small sample size, limited statistical power for detecting individual SNPs, and lack of replication in external populations make it difficult to generalize or apply the findings widely. The manuscript would also benefit from thorough language editing to address typographical and formatting errors. Overall, it presents some intriguing early observations, especially regarding X-chromosome-linked variation, but requires major revisions to improve clarity, temper interpretations, and enhance overall presentation.

Addressing these points will boost the clarity and impact of the study. A thorough proofreading is needed to fix typos and maintain consistent formatting, making it easier to read.

Below are my major comments and minor suggestions for improvement:

1. The authors present a detailed post hoc power analysis showing very low power (under 20%) for individual SNP associations, but this limitation isn’t clearly highlighted in the discussion. While using polygenic risk score aggregation boosts statistical power, it doesn’t completely address concerns about false positives at the single-marker level.

2. Excluding subclinical mastitis cases creates a phenotype definition that differs from most mastitis GWAS studies. While this approach improves clinical specificity, it also makes comparisons with previous research more difficult and may exclude genetically significant cases. The reasoning behind this decision should be explained more clearly.

3. The chosen GWAS significance threshold (–log₁₀P > 5.0) is less stringent than the traditional Bonferroni correction based on the total number of SNPs tested. It would be useful to explain this choice more clearly and acknowledge the increased risk of false-positive associations.

4. One key finding is the clustering of significant SNPs on the X chromosome. However, the current analytical framework doesn’t specifically account for sex-specific inheritance, dosage compensation, or X-chromosome inactivation. These important biological and statistical factors deserve a deeper discussion.

5. Out of the 15 top SNPs chosen for validation, only 7 were successfully confirmed. The manuscript doesn’t mention why the other SNPs failed validation, such as potential primer design problems or sequencing quality issues. Including this detail would help in evaluating the study’s robustness.

6. The functional discussion relies largely on other studies including human studies (e.g., NXPE4) and doesn’t include cattle-specific functional or expression data. While this is fine for exploratory purposes, these interpretations should be clearly marked as speculative.

7. The validation relied on animals from the same herd, reducing both independence and broader population relevance. The absence of external replication should be more clearly acknowledged as a major limitation.

Minor Comments

Introduction

Line number 61-63: Sentence ending before citation [2] lacks punctuation. Add punctuation for sentence separation.

Line number 68-77: In this section, the author should emphasize in the introduction that genetic improvements for traits with low heritability and multifactorial nature can be enhanced through genomic or genetic selection.

Line number 77: Although it was emphasized that “Despite these advancements, reducing mastitis incidence through genomic selection remains challenging”, the author failed to explain why genomic selection remains challenging.

Line number 79-93: This section overemphasizes the limitations of SCC without clearly connecting them to the study’s genomic objective. The paragraph lacks a focused articulation of the specific knowledge gap that the present study aims to address, particularly with respect to recurrent clinical mastitis. While the paragraph argues that SCC is influenced by physiological factors (parity, age, stress, production), it does not clearly link this limitation to the need for genomic markers. The reader is left knowing that SCC is imperfect, but not yet convinced why genetics, specifically SNP discovery, is the necessary solution.

Lines number 86-91 mention recurrence, but the paragraph fails to emphasize why recurrence is biologically or genetically distinct from single episodes.

Line 94-96: The author mentioned that “In this study, mastitis -susceptible cows were defined as those that experienced three or more episodes of clinical mastitis (one episode includes onset, healing, and recurrence) within the same lactation period”. I would like to raise the point that if animals experience two episodes of clinical mastitis within the same lactation period, should they not be considered mastitis-susceptible? Could you clarify the rationale behind your definition of mastitis-susceptible animals?

Materials and methods

This manuscript presents serious problems in clearly describing and providing detailed models for GWAS analysis. Moreover, it lacks several critical methodological details and raises concerns that directly affect the reproducibility, rigor, and interpretability of the findings. The GWAS and variant filtering strategy is insufficiently transparent and deviates from common best practices. Key elements are missing or unclear, including: 1) the exact statistical model used for association testing (e.g., logistic regression, chi-square, mixed model), 2) whether population structure and relatedness were explicitly corrected beyond PCA visualization (e.g., inclusion of principal components or a kinship matrix in the model), and 3) justification for using a fixed –log₁₀P > 5.0 threshold rather than a genome-wide significance level derived from the actual number of independent tests. Addressing these issues is essential to substantiate the claim that the identified SNPs reflect true genetic susceptibility to recurrent clinical mastitis rather than herd-specific or management-driven effects.

Line number 117-122: “The TMR……. (2001) (S1 Table)”. This part needs to be paraphrased and re-written it to make it more understandable.

Line 136: “The top 10% of cows by parity were excluded”. What do you mean???

Line 139: “….. mastitis: n = 43) …. Which type of mastitis????

Line number 175-176: The author noted that “…. amplification success was confirmed via 2% agarose gel electrophoresis (S1 Fig). However, this sentence should be written more professionally, as agar gel electrophoresis is used to assess the quality of the amplified DNA.

Line 170: Title should be modified as the section states about validation of selected SNPs.

Line 183-185: The assumption that each SNP explains ~7% of phenotypic variance (R² ≈ 0.07) is optimistic and not well justified, particularly for a complex disease such as mastitis. No empirical or literature-based rationale is provided to support this effect size, which may lead to inflated power estimates, especially given the small sample size (n = 50).

Line number 190-193: The description of transforming odds ratios to liability-scale R², calculating non-centrality parameters (NCPs), and aggregating them into a polygenic score is overly technical and difficult to follow.

Line number 207: “PCA module in Python”. Incomplete sentence.

Results

Line number 221-224: Please re-write this sentence to make more readable.

Line number 232: Instead of writing fat and protein concentration, I suggest using fat and protein percentage, since these parameters are typically expressed as percentages.

Line number 256-260. Did the author examine the additive (a), dominant (d), and substitution (α) effects of these 15 highly significant SNPs? If not, I suggest doing so.

Line 264-265: “Herd traits, including key milk parameters and parity, are presented in S8 Table”.

I fail to understand the importance of this sentence in this section.

Line number 266-268: The authors reported that cows with homozygous or heterozygous genotypes exhibited a higher incidence of mastitis. Why are animals with these genotypes more prone to mastitis?

Line number 290-292: The diplotype analysis requires clearer explanation and more detailed justification, as it provides no meaningful insight in its current form. This should include how and why these SNPs were selected for diplotype analysis.

Discussion

Given the low power for individual SNP detection (<20%), the Discussion should frame these findings as hypothesis-generating rather than causal. The clinical relevance of the SNP panel is overstated, as its high specificity (98%) is offset by modest sensitivity (47%), limiting its value for selection or screening. The Discussion does not address the consequences of missing over half of susceptible cows, fails to compare this trade-off with existing breeding tools, and overlooks potential confounding between genetic susceptibility, infection persistence, and management-related recurrence.

Line number 312-313: This section fails to describe how the association analysis was performed and how it was selected for validation.

Line number 330-332: This discussion section refers to human studies, which may bias the interpretation when applied to bovines.

Line number 360-363: “Our results…..in dairy cattle.” This part needs to be supported and justified with relevant literature; otherwise, it remains a black box.

Line number 360-363: Is there any previous studies / reports with similar arguments???

Conclusions and recommendations

The authors attempted to highlight the limitations of this study; however, these limitations are not effectively integrated into the Discussion in relation to the significant SNPs. I strongly recommend strengthening the Discussion by explicitly incorporating the mentioned limitations.

References

The authors are solely responsible for following the journal’s writing guidelines for references.

Reviewer #2: Note to the authors:

This paper describes a GWAS analysis to identify mastitis markers that can aid herd management and selection. They start with providing a definition of mastitis-susceptibility. They utilized 50 genome sequences to filter SNP candidates, and used another set of 100 animals to validate the association. They propose 7 SNP markers to be combined in sets of 2-3 markers to screen with confidence for animals with genetic predisposition to mastitis. This study deserves to be published and can benefit the dairy industry.

I recommend to ACCEPT this paper for publication to Plos ONE.

Minor comments are to improve the readability of the paper. I suggest the following:

CONCLUSION

Line 391 - 395

I suggest to transfer the 'In conclusion' phrase to the second sentence. To read: "In conclusion, this study suggest a contribution of X-linked genetic variation to mastitis

susceptibility and provide preliminary markers that may aid in the development of

genomic selection panels."

RESULTS

All figure titles (Fig 1 -6) could be improved by adding short description of the results. It is a general rule that the tables and figures be able to stand-alone (meaning, without need for reading the body of the manuscript). Thus, all details are included especially a short description of the result. Here are examples:

In Figure 2. Principal component analysis (PCA) of SNP data showed no significant differences in SNP distribution between healthy and mastitis group.

In Figure 3. Genome-wide association analysis identified 15 highly associated SNPs for further validation.

In Figure 1 - please indicate how many animals were sequenced? Also indicate this data in Materials and Methods.

Table titles also need to be improved for better readability.

Table 3. Include number of animals in the footnote. "Based from 100 cows."

Table 2. Seven candidate SNP markers associated with mastitis susceptibility were identified by genome-wide association analysis. Also, Please include a row in Table 2 showing allele frequency distribution from the sequencing result of 100 animals.

DISCUSSION

Line 362 - it is best to keep discussion within the scope of the study, thus, specify mastitis. to read: "a crucial role in determining mastitis immune response"

Line 365: indicate the meaning of lower p-values, to read: allowing us to identify variants with lower p-values (highly significant).

Line 352: same comment: "high p-values (low significance)"

Line 325: I suggest this revision: "for the early identification of cows with genetic predisposition to recurrent mastitis."

Line 318: Did you mean heterozygote and homozygote mutant genotype? I suggest this revision: "cows carrying a mutant allele were considered"

Line 307: I suggest this revision: "false positives in traditional susceptibility screening"

MATERIALS AND METHODS

For those who do not understand sensitivity and specificity scores, it would be nice to add a Supplementary table showing the 2x2 contingency table or confusion matrix how you arrive to those values, for each SNP. Also add a 1 line discussion on the relevance of the kappa coefficient.

Please add a line or related articles justifying that a significance threshold of p-value< 1x10^-6 is sufficient for whole genome-based SNPs? It is definitely higher than Kurz et al 2019 which set it at 1x10^-4 but which also used a SNP Chip BovineHD, but industry standard is supposedly set at 1x10^-8. See ref: https://doi.org/10.1093/genetics/iyaf056

Line 171 ... "were validated by PCR and Sanger sequencing."

Bos taurus and ad libitum should be italicized.

ABSTRACT

Line 41 should include mastitis, to read "mastitis immune response..". Best to avoid over generalization.

**********

6. PLOS authors have the option to publish the peer review history of their article (what does this mean?). If published, this will include your full peer review and any attached files.

If you choose “no”, your identity will remain anonymous but your review may still be made public.

**Do you want your identity to be public for this peer review?**  For information about this choice, including consent withdrawal, please see our Privacy Policy.

Reviewer #1: **Yes:**  Dr. Destaw Worku

Reviewer #2: No

**********

[NOTE: If reviewer comments were submitted as an attachment file, they will be attached to this email and accessible via the submission site. Please log into your account, locate the manuscript record, and check for the action link "View Attachments". If this link does not appear, there are no attachment files.]

To ensure your figures meet our technical requirements, please review our figure guidelines: https://journals.plos.org/plosone/s/figures

You may also use PLOS’s free figure tool, NAAS, to help you prepare publication quality figures: https://journals.plos.org/plosone/s/figures#loc-tools-for-figure-preparation.

NAAS will assess whether your figures meet our technical requirements by comparing each figure against our figure specifications.

Attachment Submitted filename: Review Report.docx

# Author comment: Author response to Decision Letter 1

**DOI:** 10.1371/journal.pone.0355230.r002

23 Mar 2026

Response to Editor:

Comment#1

- Please ensure that your manuscript meets PLOS ONE's style requirements, including those for file naming. The PLOS ONE style templates can be found at https://journals.plos.org/plosone/s/file?id=wjVg/PLOSOne_formatting_sample_main_body.pdf and

https://journals.plos.org/plosone/s/file?id=ba62/PLOSOne_formatting_sample_title_authors_affiliations.pdf

[Response]

We thank the editor for this important comment. We have carefully revised the manuscript to ensure full compliance with PLOS ONE’s style requirements, including formatting and file naming in accordance with provided templates.

In addition to the revisions requested by the reviewers, we corrected an error in Table 4: the SNP combination previously listed as "SNP1+SNP2" has been corrected to "SNP1+SNP7" to accurately reflect the actual combination analyzed.

Comment#2

- We noticed you have some minor occurrence of overlapping text with the following previous publication(s), which needs to be addressed:

https://www.cell.com/heliyon/fulltext/S2405-8440(24)05820-1?_returnURL=https%3A%2F%2Flinkinghub.elsevier.com%2Fretrieve%2Fpii%2FS2405844024058201%3Fshowall%3Dtrue

In your revision ensure you cite all your sources (including your own works), and quote or rephrase any duplicated text outside the methods section. Further consideration is dependent on these concerns being addressed.

[Response]

We thank the editor for this important comment. We carefully reviewed the manuscript and identified overlapping text, particularly in the Material and Methods section in relation to our previous publication. Because the experimental design and animal cohort were similar to those used in our previous study, partial similarity in methodological description was unavoidable. However, the relevant sections have been thoroughly revised by rephrasing and restructuring the text to eliminate duplication. In addition, our previous study has now been appropriately cited where relevant. We confirm that all duplicated text has been addressed and that appropriate citations have been included. Please see the lines 110-113, 116-125, 128-139, 142-160.

Comment#3

- PLOS ONE now requires that authors provide the original uncropped and unadjusted images underlying all blot or gel results reported in a submission’s figures or Supporting Information files. This policy and the journal’s other requirements for blot/gel reporting and figure preparation are described in detail at https://journals.plos.org/plosone/s/figures#loc-blot-and-gel-reporting-requirements and https://journals.plos.org/plosone/s/figures#loc-preparing-figures-from-image-files. When you submit your revised manuscript, please ensure that your figures adhere fully to these guidelines and provide the original underlying images for all blot or gel data reported in your submission. See the following link for instructions on providing the original image data: https://journals.plos.org/plosone/s/figures#loc-original-images-for-blots-and-gels.

In your cover letter, please note whether your blot/gel image data are in Supporting Information or posted at a public data repository, provide the repository URL if relevant, and provide specific details as to which raw blot/gel images, if any, are not available. Email us at plosone@plos.org if you have any questions.

[Response]

We appreciate the editor’s helpful comment. We confirm that the original uncropped and unadjusted gel images corresponding to Fig.4 have been provided in the Supporting Information (S1). The corresponding figure legends have been revised to clearly indicate that these images represent the original data underlying the processed figures shown in the main manuscript.

Comment#4

- Thank you for stating the following financial disclosure:

“This study was supported by the Livestock Promotional Subsidies of the Japan Racing Association (JRA) (K.N.).”

Please state what role the funders took in the study. If the funders had no role, please state: "The funders had no role in study design, data collection and analysis, decision to publish, or preparation of the manuscript."

If this statement is not correct you must amend it as needed.

Please include this amended Role of Funder statement in your cover letter; we will change the online submission form on your behalf.

[Response]

We thank the editor for this important comment. The funding statement has been revised to clarify the role of the funder. The funders had no role in study design, data collection and analysis, decision to publish, or preparation of the manuscript.

Comment#5

- Please note that funding information should not appear in any section or other areas of your manuscript. We will only publish funding information present in the Funding Statement section of the online submission form. Please remove any funding-related text from the manuscript.

[Response]

We thank the editor for this important comment. All funding-related information has been removed from the manuscript text and is now reported only in the Funding Statement section of the online submission form, in accordance with PLOS ONE guidelines.

Comment#6

-Thank you for uploading your study's underlying data set. Unfortunately, the repository you have noted in your Data Availability statement does not qualify as an acceptable data repository according to PLOS's standards.

At this time, please upload the minimal data set necessary to replicate your study's findings to a stable, public repository (such as figshare or Dryad) and provide us with the relevant URLs, DOIs, or accession numbers that may be used to access these data. For a list of recommended repositories and additional information on PLOS standards for data deposition, please see https://journals.plos.org/plosone/s/recommended-repositories.

[Response]

We thank the editor for this comment. In response, we have deposited the minimal dataset necessary to replicate the findings of this study in figshare, which is listed among PLOS ONE's recommended repositories. The dataset is publicly available under the following DOI: https://doi.org/10.6084/m9.figshare.31829746. In addition, the variant call data remain available through the European Variation Archive (EVA) at EMBL-EBI under project accession number PRJEB88141. The Data Availability statement has been revised accordingly to include the repository name, accession information, and direct URL.

Comment#7

- In the online submission form you indicate that your data is not available for proprietary reasons and have provided a contact point for accessing this data. Please note that your current contact point is a co-author on this manuscript. According to our Data Policy, the contact point must not be an author on the manuscript and must be an institutional contact, ideally not an individual. Please revise your data statement to a non-author institutional point of contact, such as a data access or ethics committee, and send this to us via return email. Please also include contact information for the third party organization, and please include the full citation of where the data can be found.

[Response]

We thank the editor for this comment. As described in our response to Comment #6, the data underlying this study have now been deposited in a public repository figshare and are freely accessible without restriction. Because the data are now publicly available, a third-party institutional contact point for data access is no longer required. The Data Availability statement has been updated accordingly.

Comment#8

- If the reviewer comments include a recommendation to cite specific previously published works, please review and evaluate these publications to determine whether they are relevant and should be cited. There is no requirement to cite these works unless the editor has indicated otherwise.

[Response]

We appreciate the editor’s comment. We have reviewed the suggested references and determined that they are not directly relevant to the present study.

Comment#9

- Please review your reference list to ensure that it is complete and correct. If you have cited papers that have been retracted, please include the rationale for doing so in the manuscript text, or remove these references and replace them with relevant current references. Any changes to the reference list should be mentioned in the rebuttal letter that accompanies your revised manuscript. If you need to cite a retracted article, indicate the article’s retracted status in the References list and also include a citation and full reference for the retraction notice.

[Response]

We appreciate the editor’s comment. We have carefully reviewed the reference list to ensure that all citations are accurate and complete. No retracted articles were cited in this study. Any necessary corrections have been made in the revised manuscript.

Response to reviewer1:

General comments

Comment#1

- The authors investigate potential SNP markers associated with recurrent clinical mastitis in Holstein cattle using whole-genome resequencing followed by validation. Mastitis remains a major economic and welfare concern in dairy farming, so identifying genetic markers for susceptibility is both timely and valuable. By focusing on recurrent cases within a single lactation, the study captures a more consistent and severe phenotype compared to research based solely on SCC measurements.

[Response]

We appreciate the reviewer’s positive evaluation and recognition of the significance of our study.

Comment#2

- The study presents potentially interesting exploratory findings, particularly regarding X-linked SNPs and recurrent clinical mastitis, and while the authors acknowledge several limitations, some of their interpretations and conclusions stretch beyond what the data can truly support. The small sample size, limited statistical power for detecting individual SNPs, and lack of replication in external populations make it difficult to generalize or apply the findings widely. The manuscript would also benefit from thorough language editing to address typographical and formatting errors. Overall, it presents some intriguing early observations, especially regarding X-chromosome-linked variation, but requires major revisions to improve clarity, temper interpretations, and enhance overall presentation.

[Response]

Thank you so much for your thoughtful comments and review of the manuscript. We acknowledge the limitations of the study, including the relatively small sample size, limited statistical power, and lack of external validation. In response, we have revised the manuscript to provide a more cautious interpretation of the results and to clearly emphasize these limitations. In addition, we have carefully improved the clarity of the manuscript, including language editing and formatting corrections, to enhance overall readability and presentation.

Comment#3

- Addressing these points will boost the clarity and impact of the study. A thorough proofreading is needed to fix typos and maintain consistent formatting, making it easier to read.

[Response]

We appreciate the reviewer’s comment. We have carefully proofread the manuscript and corrected typographical errors and formatting inconsistencies to improve clarity and readability.

Major comments

Comment#1

- The authors present a detailed post hoc power analysis showing very low power (under 20%) for individual SNP associations, but this limitation isn’t clearly highlighted in the discussion. While using polygenic risk score aggregation boosts statistical power, it doesn’t completely address concerns about false positives at the single-marker level.

[Response]

Thank you so much for your important comment. We acknowledge that the statistical power for individual SNP associations was limited, as indicated by the post hoc power analysis. In response, we have revised the Discussion section to clearly highlight this limitation and to explicitly address the potential for false positives at the single-marker level. Please see the lines 495-510.

Comment#2

- Excluding subclinical mastitis cases creates a phenotype definition that differs from most mastitis GWAS studies. While this approach improves clinical specificity, it also makes comparisons with previous research more difficult and may exclude genetically significant cases. The reasoning behind this decision should be explained more clearly.

[Response]

We thank the reviewer’s important comment. We have clarified the rationale for excluding subclinical mastitis case in the manuscript. Specifically, we aimed to focus on clearly defined clinical phenotypes to improve phenotypic consistency. We have added a statement in the Materials and Methods section to explain this definition, and we have also added a statement in the Case Definition and Discussion to acknowledge that this approach may limit comparability with previous studies and may exclude genetically relevant cases. Please see the lines 134-137, 507-510.

Comment#3

- The chosen GWAS significance threshold (–log₁₀P > 5.0) is less stringent than the traditional Bonferroni correction based on the total number of SNPs tested. It would be useful to explain this choice more clearly and acknowledge the increased risk of false-positive associations.

[Response]

We appreciate the reviewer’s important comment. We have clarified the rationale for the chosen GWAS significance threshold in the Materials and Methods section. In addition, we have added a statement in the Discussion to acknowledge that this threshold is less stringent than a Bonferroni correction and may increase the risk of false positive associations. Please see the lines 503-510.

Comment#4

- One key finding is the clustering of significant SNPs on the X chromosome. However, the current analytical framework doesn’t specifically account for sex-specific inheritance, dosage compensation, or X-chromosome inactivation. These important biological and statistical factors deserve a deeper discussion.

[Response]

We appreciate the reviewer’s important comment. We acknowledge that the interpretation of X-linked SNP clustering is currently based on limited analytical considerations. We have revised the Discussion to clarify that these findings are preliminary and speculative, and to emphasize the need for further studies incorporating sex-specific inheritance, dosage compensation, and X-chromosome inactivation. Please see the lines 490-494.

Comment#5

-Out of the 15 top SNPs chosen for validation, only 7 were successfully confirmed. The manuscript doesn’t mention why the other SNPs failed validation, such as potential primer design problems or sequencing quality issues. Including this detail would help in evaluating the study’s robustness.

[Response]

we acknowledge that not all candidate SNPs were successfully validated. This was primarily due to technical limitations, including difficulties in primer design and sequencing quality issues. As reviewer requested, we have added a statement in the Discussion to clarify this point. Please see the lines 505-510.

Comment#6

- The functional discussion relies largely on other studies including human studies (e.g., NXPE4) and doesn’t include cattle-specific functional or expression data. While this is fine for exploratory purposes, these interpretations should be clearly marked as speculative.

[Response]

We appreciate the reviewer’s important comment. We acknowledge that the functional interpretation of candidate genes was primarily based on studies in other species. In response, we have revised the manuscript to clearly indicate that these interpretations are speculative and require further validation in cattle in Discussion. Please see the lines 440-441.

Comment#7

-The validation relied on animals from the same herd, reducing both independence and broader population relevance. The absence of external replication should be more clearly acknowledged as a major limitation.

[Response]

We appreciate the reviewer’s valuable comment. We acknowledge that the validation was conducted using animals from the same herd, which may limit independence and generalizability. We have added a statement in the Discussion to clearly highlight this as a limitation. Please see the lines 512-518.

Minor

Attachment Submitted filename: Response Final .docx

# Peer review: Decision Letter 1

**DOI:** 10.1371/journal.pone.0355230.r003

**Authors:** Pierre Germon

10 Jun 2026

PONE-D-25-49503R1

Exploratory identification of candidate SNP markers associated with recurrent clinical mastitis in Holstein cattle

PLOS One

Dear Dr. Nagaoka,

Thank you for submitting your manuscript to PLOS ONE. After careful consideration, we feel that it has merit but does not fully meet PLOS ONE’s publication criteria as it currently stands. Therefore, we invite you to submit a revised version of the manuscript that addresses the points raised during the review process.

Althought the findings are interesting and raise interesting questions regarding association of X-linked snps with recurent mastitis, comments raised on the methods and limits due to low sample size should be addressed.

If data are available, it would also be interesting to more precisely define the mastitis cases by indicating which pathogen was responsible for mastitis cases recorded.

Please submit your revised manuscript by Jul 25 2026 11:59PM. If you will need more time than this to complete your revisions, please reply to this message or contact the journal office at plosone@plos.org. When you're ready to submit your revision, log on to https://www.editorialmanager.com/pone/ and select the 'Submissions Needing Revision' folder to locate your manuscript file.

Please include the following items when submitting your revised manuscript:

- A letter that responds to each point raised by the academic editor and reviewer(s). You should upload this letter as a separate file labeled 'Response to Reviewers'.
- A marked-up copy of your manuscript that highlights changes made to the original version. You should upload this as a separate file labeled 'Revised Manuscript with Track Changes'.
- An unmarked version of your revised paper without tracked changes. You should upload this as a separate file labeled 'Manuscript'.

If you would like to make changes to your financial disclosure, please include your updated statement in your cover letter. Guidelines for resubmitting your figure files are available below the reviewer comments at the end of this letter.

If applicable, we recommend that you deposit your laboratory protocols in protocols.io to enhance the reproducibility of your results. Protocols.io assigns your protocol its own identifier (DOI) so that it can be cited independently in the future. For instructions see: https://journals.plos.org/plosone/s/submission-guidelines#loc-laboratory-protocols. Additionally, PLOS ONE offers an option for publishing peer-reviewed Lab Protocol articles, which describe protocols hosted on protocols.io. Read more information on sharing protocols at https://plos.org/protocols?utm_medium=editorial-email&utm_source=authorletters&utm_campaign=protocols.

As the corresponding author, your ORCID iD is verified in the submission system and will appear in the published article. PLOS supports the use of ORCID, and we encourage all coauthors to register for an ORCID iD and use it as well. Please encourage your coauthors to verify their ORCID iD within the submission system before final acceptance, as unverified ORCID iDs will not appear in the published article. Only the individual author can complete the verification step; PLOS staff cannot verify ORCID iDs on behalf of authors.

We look forward to receiving your revised manuscript.

Kind regards,

Pierre Germon

Academic Editor

PLOS One

Journal Requirements:

If the reviewer comments include a recommendation to cite specific previously published works, please review and evaluate these publications to determine whether they are relevant and should be cited. There is no requirement to cite these works unless the editor has indicated otherwise.

Please review your reference list to ensure that it is complete and correct. If you have cited papers that have been retracted, please include the rationale for doing so in the manuscript text, or remove these references and replace them with relevant current references. Any changes to the reference list should be mentioned in the rebuttal letter that accompanies your revised manuscript. If you need to cite a retracted article, indicate the article’s retracted status in the References list and also include a citation and full reference for the retraction notice.

[Note: HTML markup is below. Please do not edit.]

Reviewers' comments:

Reviewer's Responses to Questions

**Comments to the Author**

1. If the authors have adequately addressed your comments raised in a previous round of review and you feel that this manuscript is now acceptable for publication, you may indicate that here to bypass the “Comments to the Author” section, enter your conflict of interest statement in the “Confidential to Editor” section, and submit your "Accept" recommendation.

Reviewer #1: All comments have been addressed

Reviewer #3: (No Response)

**********

2. Is the manuscript technically sound, and do the data support the conclusions?

The manuscript must describe a technically sound piece of scientific research with data that supports the conclusions. Experiments must have been conducted rigorously, with appropriate controls, replication, and sample sizes. The conclusions must be drawn appropriately based on the data presented.

Reviewer #1: Yes

Reviewer #3: Partly

**********

3. Has the statistical analysis been performed appropriately and rigorously?

Reviewer #1: Yes

Reviewer #3: No

**********

4. Have the authors made all data underlying the findings in their manuscript fully available?

The PLOS Data policy requires authors to make all data underlying the findings described in their manuscript fully available without restriction, with rare exception (please refer to the Data Availability Statement in the manuscript PDF file). The data should be provided as part of the manuscript or its supporting information, or deposited to a public repository. For example, in addition to summary statistics, the data points behind means, medians and variance measures should be available. If there are restrictions on publicly sharing data—e.g. participant privacy or use of data from a third party—those must be specified.

Reviewer #1: Yes

Reviewer #3: Yes

**********

5. Is the manuscript presented in an intelligible fashion and written in standard English?

PLOS ONE does not copyedit accepted manuscripts, so the language in submitted articles must be clear, correct, and unambiguous. Any typographical or grammatical errors should be corrected at revision, so please note any specific errors here.

Reviewer #1: Yes

Reviewer #3: Yes

**********

6. Review Comments to the Author

Please use the space provided to explain your answers to the questions above. You may also include additional comments for the author, including concerns about dual publication, research ethics, or publication ethics. (Please upload your review as an attachment if it exceeds 20,000 characters)

Reviewer #1: The revised manuscript has satisfactorily addressed the concerns raised in the previous review round, and I recommend it for publication.

Reviewer #3: The revised manuscript has improved in clarity and presentation, and I appreciate the authors’ efforts to address several of the previous comments. In particular, the manuscript now provides clearer information on the case definition of recurrent clinical mastitis, acknowledges the exploratory nature of the study more explicitly, and includes additional discussion of limitations related to sample size and validation. However, I still have several methodological concerns that should be addressed before the manuscript can be considered for acceptance.

My main concern remains the GWAS analytical framework. Although the authors clarified that CLC Genomics Workbench was used for the GWAS, the issue is not the use of the software itself, but whether the specific case–control chi-square association test is sufficient for this dataset. Recurrent clinical mastitis is a complex and multifactorial disease trait influenced by genetic, physiological, environmental, and management-related factors. Given the small sample size, same-herd design, and potential cryptic relatedness among animals, a simple chi-square test may not adequately control false-positive associations.

If the authors wish to retain this analytical framework, they should provide stronger justification and, ideally, cite studies where CLC Genomics Workbench was specifically used for GWAS association testing (livestock research), not only for read quality control, mapping, or variant calling. Alternatively, the authors should consider reanalyzing the data using a mixed-model GWAS framework that can account for relatedness, population structure, and relevant covariates. For example, depending on the available pedigree, genotype, and phenotype data, software commonly used in animal breeding and genomic evaluation, such as the BLUPF90 family of programs, could be considered. Since recurrent mastitis may be treated either as a binary disease phenotype or as a count trait based on the number of episodes, the statistical model should be selected accordingly.

The authors state that no additional correction for population structure was applied because all animals originated from a single herd and no clear stratification was observed in the PCA plot. However, PCA visualization alone is not sufficient to exclude cryptic relatedness or subtle family structure, particularly in a small Holstein dataset. Animals within the same herd may still be related through common sires, dams, or breeding lines. A genomic relationship or kinship matrix can be estimated from genome-wide SNP data and incorporated into a mixed-model GWAS to account for pairwise relatedness among animals. The authors should either apply such correction or explicitly acknowledge the lack of kinship correction as a major limitation.

The statement regarding Bonferroni correction and the –log₁₀(P) threshold should also be clarified. In the Materials and Methods section, the statement in lines 186–189 should be moved to the Statistical Analysis section, as this pertains to GWAS association testing rather than variant calling or annotation. More importantly, the threshold of –log₁₀(P) > 5.0 does not correspond to a Bonferroni-corrected genome-wide significance threshold for the 536,184 SNPs retained after quality control. If the authors used –log₁₀(P) > 5.0 as an exploratory threshold to prioritize candidate SNPs, this should be stated explicitly. I suggest removing the claim that Bonferroni correction was applied unless the authors provide the actual Bonferroni-corrected threshold and explain how it was used in the analysis.

The Q-Q plot should also be interpreted more cautiously. Several livestock GWAS studies routinely report the genomic inflation factor, λ, together with Q-Q plots to evaluate whether association statistics are inflated by population structure, relatedness, or model misspecification. I suggest that the authors report λ for their GWAS results. This is particularly important because the Q-Q plot appears to show some upward deviation, and the current analysis did not include a kinship matrix or principal components as covariates. Reporting λ would help readers assess whether the chi-square association test was adequately calibrated.

The wording regarding SNP effects should be revised. In line 262, the statement that “each individual SNP explained approximately 7% of the phenotypic variance” may be misleading because it implies that this value was empirically estimated from the data. However, based on the Methods section, the 7% value appears to have been used as an assumed or illustrative effect size for the statistical power calculation. The authors should revise this wording to clearly indicate that R² ≈ 0.07 was an assumption used for power analysis, not an observed estimate of phenotypic variance explained by each SNP. Alternatively, this statement should be removed if the authors cannot provide an empirical basis for the 7% estimate.

The validation analysis also requires clarification. The authors should clearly state whether the phenotype analyzed in the validation step was mastitis status or the number of mastitis episodes. If the outcome was mastitis incidence/status, logistic regression, chi-square test, or Fisher’s exact test would be more appropriate. If the outcome was the number of mastitis episodes, as shown in the figure, then the trait is count data; therefore, Poisson regression or negative binomial regression would be more suitable than Kruskal–Wallis, particularly if overdispersion is present. The authors should justify the statistical test used and report effect estimates with confidence intervals and adjusted P-values where possible.

I also suggest replacing the term “significant SNPs” with more cautious wording such as “candidate SNPs,” “putative SNPs,” or “putative mastitis-associated SNPs.” Given the exploratory threshold, limited sample size, same-herd validation, and lack of external independent replication, the current wording may overstate the strength of evidence. Similarly, terms such as “validated markers,” “predictive panel,” and “marker-assisted selection” should be softened unless supported by stronger statistical evidence and external validation.

In summary, the manuscript has improved, but several important methodological issues remain unresolved. I recommend major revision.

**********

7. PLOS authors have the option to publish the peer review history of their article (what does this mean?). If published, this will include your full peer review and any attached files.

If you choose “no”, your identity will remain anonymous but your review may still be made public.

**Do you want your identity to be public for this peer review?**  For information about this choice, including consent withdrawal, please see our Privacy Policy.

Reviewer #1: **Yes:**  Destaw Worku Mengistu, Bahir Dar University, Department of Animal Sciences

Reviewer #3: No

**********

[NOTE: If reviewer comments were submitted as an attachment file, they will be attached to this email and accessible via the submission site. Please log into your account, locate the manuscript record, and check for the action link "View Attachments". If this link does not appear, there are no attachment files.]

To ensure your figures meet our technical requirements, please review our figure guidelines: https://journals.plos.org/plosone/s/figures

You may also use PLOS’s free figure tool, NAAS, to help you prepare publication quality figures: https://journals.plos.org/plosone/s/figures#loc-tools-for-figure-preparation.

NAAS will assess whether your figures meet our technical requirements by comparing each figure against our figure specifications.

# Author comment: Author response to Decision Letter 2

**DOI:** 10.1371/journal.pone.0355230.r004

2 Jul 2026

Editor request

If data are available, it would also be interesting to more precisely define the mastitis cases by indicating which pathogen was responsible for mastitis cases recorded.

Reply:

We thank the editor for this suggestion. Bacteriological culture data were available for the 25 mastitis-susceptible cows in the discovery cohort. The most frequently isolated pathogens were coagulase-negative staphylococci (CNS; 15/25 cows), Escherichia coli (12/25), Streptococcus spp. (12/25), and Klebsiella spp. (11/25). Notably, 21 of 25 cows experienced mastitis caused by multiple different pathogen species across episodes, suggesting that the recurrent mastitis phenotype in these animals is more likely attributable to host-related susceptibility factors than to persistent infection by a single pathogen. These data have been added to the Results section and a detailed summary is provided in new S7 Table. Please see lines 293-299.

To Reviewer3

My main concern remains the GWAS analytical framework. Although the authors clarified that CLC Genomics Workbench was used for the GWAS, the issue is not the use of the software itself, but whether the specific case–control chi-square association test is sufficient for this dataset. Recurrent clinical mastitis is a complex and multifactorial disease trait influenced by genetic, physiological, environmental, and management-related factors. Given the small sample size, same-herd design, and potential cryptic relatedness among animals, a simple chi-square test may not adequately control false-positive associations.

If the authors wish to retain this analytical framework, they should provide stronger justification and, ideally, cite studies where CLC Genomics Workbench was specifically used for GWAS association testing (livestock research), not only for read quality control, mapping, or variant calling. Alternatively, the authors should consider reanalyzing the data using a mixed-model GWAS framework that can account for relatedness, population structure, and relevant covariates. For example, depending on the available pedigree, genotype, and phenotype data, software commonly used in animal breeding and genomic evaluation, such as the BLUPF90 family of programs, could be considered. Since recurrent mastitis may be treated either as a binary disease phenotype or as a count trait based on the number of episodes, the statistical model should be selected accordingly.

Reply:

We acknowledge the reviewer's concern. To address this, we performed GLMM analyses including sire as a random effect. All seven candidate SNPs remained significantly associated with mastitis status (S12 Table). Poisson mixed-effects models for mastitis episode count also confirmed significant associations for all SNPs (S12 Table). The estimated sire variance was negligible, supporting the robustness of the identified associations. Complete pedigree information for a full mixed-model GWAS (e.g., BLUPF90) was not available, which is acknowledged as a limitation. Please see the Materials and Methods and Results sections. Please see lines 260-268, 412-420.

The authors state that no additional correction for population structure was applied because all animals originated from a single herd and no clear stratification was observed in the PCA plot. However, PCA visualization alone is not sufficient to exclude cryptic relatedness or subtle family structure, particularly in a small Holstein dataset. Animals within the same herd may still be related through common sires, dams, or breeding lines. A genomic relationship or kinship matrix can be estimated from genome-wide SNP data and incorporated into a mixed-model GWAS to account for pairwise relatedness among animals. The authors should either apply such correction or explicitly acknowledge the lack of kinship correction as a major limitation.

Reply:

We agree that PCA alone cannot exclude cryptic relatedness. To address this, we obtained sire information for all 100 cows and confirmed that the two largest sire families contained both healthy and mastitis cows, with no obvious clustering of mastitis cases (S2 Fig). Furthermore, the GLMM analyses described above (Comment 1) included sire as a random effect, and the estimated sire variance was negligible. Although a full genomic relationship matrix could not be constructed from the available data, the lack of kinship correction in the primary GWAS has been acknowledged as a limitation in the Discussion. Please see lines 535-540.

The statement regarding Bonferroni correction and the –log₁₀(P) threshold should also be clarified. In the Materials and Methods section, the statement in lines 186–189 should be moved to the Statistical Analysis section, as this pertains to GWAS association testing rather than variant calling or annotation. More importantly, the threshold of –log₁₀(P) > 5.0 does not correspond to a Bonferroni-corrected genome-wide significance threshold for the 536,184 SNPs retained after quality control. If the authors used –log₁₀(P) > 5.0 as an exploratory threshold to prioritize candidate SNPs, this should be stated explicitly. I suggest removing the claim that Bonferroni correction was applied unless the authors provide the actual Bonferroni-corrected threshold and explain how it was used in the analysis.

Reply:

We agree with the reviewer. The –log₁₀(P) > 5.0 threshold was used as an exploratory threshold to prioritize candidate SNPs, not as a Bonferroni-corrected genome-wide significance level. We have removed the claim of Bonferroni correction, clarified the exploratory nature of the threshold, and moved the relevant statement from the Sequence Data Analysis section to the Statistical Analysis section. Please see lines 254-259.

The Q-Q plot should also be interpreted more cautiously. Several livestock GWAS studies routinely report the genomic inflation factor, λ, together with Q-Q plots to evaluate whether association statistics are inflated by population structure, relatedness, or model misspecification. I suggest that the authors report λ for their GWAS results. This is particularly important because the Q-Q plot appears to show some upward deviation, and the current analysis did not include a kinship matrix or principal components as covariates. Reporting λ would help readers assess whether the chi-square association test was adequately calibrated.

Reply:

We thank the reviewer for this suggestion. The present study was designed as an exploratory WGS-based candidate variant identification study rather than a conventional genome-wide association study. Therefore, λ was not calculated in the original analysis pipeline. In addition, the archived association results contained only variants that passed significance filtering, and the complete genome-wide P-value distribution was not retained for retrospective analysis. To directly address the reviewer's concern regarding potential confounding by relatedness, we performed pedigree-based mixed-effects analyses. GLMMs with sire as a random effect confirmed that all seven candidate SNPs remained significantly associated with mastitis status, and Poisson mixed-effects models yielded consistent results for mastitis episode counts (S12 Table). The estimated sire variance was negligible, indicating that the observed associations are robust to familial relatedness. We have acknowledged the inability to report λ as a limitation in the Discussion. Please see lines 540-543.

The wording regarding SNP effects should be revised. In line 262, the statement that “each individual SNP explained approximately 7% of the phenotypic variance” may be misleading because it implies that this value was empirically estimated from the data. However, based on the Methods section, the 7% value appears to have been used as an assumed or illustrative effect size for the statistical power calculation. The authors should revise this wording to clearly indicate that R² ≈ 0.07 was an assumption used for power analysis, not an observed estimate of phenotypic variance explained by each SNP. Alternatively, this statement should be removed if the authors cannot provide an empirical basis for the 7% estimate.

Reply:

We agree that the original wording was misleading. We have revised the statement to clarify that R² ≈ 0.07 was an assumed effect size used for statistical power estimation, not an empirically observed value. Please see the Results section. Please see lines 272-276.

The validation analysis also requires clarification. The authors should clearly state whether the phenotype analyzed in the validation step was mastitis status or the number of mastitis episodes. If the outcome was mastitis incidence/status, logistic regression, chi-square test, or Fisher’s exact test would be more appropriate. If the outcome was the number of mastitis episodes, as shown in the figure, then the trait is count data; therefore, Poisson regression or negative binomial regression would be more suitable than Kruskal–Wallis, particularly if overdispersion is present. The authors should justify the statistical test used and report effect estimates with confidence intervals and adjusted P-values where possible.

Reply:

We thank the reviewer for this clarification. In the original validation analysis, the Kruskal–Wallis test was used as a non-parametric approach to compare mastitis episode counts across genotype groups (Fig. 6). To address the reviewer's concern, we performed additional analyses using GLMM with mastitis status as a binary outcome and a Poisson mixed-effects model with mastitis episode count as a count outcome, both including sire as a random effect (S12 Table). These analyses confirmed significant associations for all seven candidate SNPs. The Methods sections have been updated accordingly. Please see lines 243-247.

I also suggest replacing the term “significant SNPs” with more cautious wording such as “candidate SNPs,” “putative SNPs,” or “putative mastitis-associated SNPs.” Given the exploratory threshold, limited sample size, same-herd validation, and lack of external independent replication, the current wording may overstate the strength of evidence. Similarly, terms such as “validated markers,” “predictive panel,” and “marker-assisted selection” should be softened unless supported by stronger statistical evidence and external validation.

Reply:

We agree with the reviewer. Throughout the manuscript, we have replaced "significant SNPs" with "candidate SNPs" or "putative mastitis-associated SNPs," softened "validated markers" to "candidate markers evaluated in the validation cohort," replaced "predictive panel" with "candidate marker panel," and used "marker-assisted selection" only in the context of future prospects. These changes have been applied consistently throughout the Abstract, Results, and Discussion.

Attachment Submitted filename: Response .docx

# Peer review: Decision Letter 2

**DOI:** 10.1371/journal.pone.0355230.r005

**Authors:** Pierre Germon

19 Jul 2026

Exploratory identification of candidate SNP markers associated with recurrent clinical mastitis in Holstein cattle

PONE-D-25-49503R2

Dear Dr. Nagaoka,

We’re pleased to inform you that your manuscript has been judged scientifically suitable for publication and will be formally accepted for publication once it meets all outstanding technical requirements.

Within one week, you’ll receive an e-mail detailing the required amendments. When these have been addressed, you’ll receive a formal acceptance letter and your manuscript will be scheduled for publication.

An invoice will be generated when your article is formally accepted. Please note, if your institution has a publishing partnership with PLOS and your article meets the relevant criteria, all or part of your publication costs will be covered. Please make sure your user information is up-to-date by logging into Editorial Manager at Editorial Manager® and clicking the ‘Update My Information' link at the top of the page. For questions related to billing, please contact billing support.

If your institution or institutions have a press office, please notify them about your upcoming paper to help maximize its impact. If they’ll be preparing press materials, please inform our press team as soon as possible -- no later than 48 hours after receiving the formal acceptance. Your manuscript will remain under strict press embargo until 2 pm Eastern Time on the date of publication. For more information, please contact onepress@plos.org.

Kind regards,

Pierre Germon

Academic Editor

PLOS One

Additional Editor Comments (optional):

Reviewers' comments:

Reviewer's Responses to Questions

**Comments to the Author**

1. If the authors have adequately addressed your comments raised in a previous round of review and you feel that this manuscript is now acceptable for publication, you may indicate that here to bypass the “Comments to the Author” section, enter your conflict of interest statement in the “Confidential to Editor” section, and submit your "Accept" recommendation.

Reviewer #3: All comments have been addressed

Reviewer #4: All comments have been addressed

**********

2. Is the manuscript technically sound, and do the data support the conclusions?

The manuscript must describe a technically sound piece of scientific research with data that supports the conclusions. Experiments must have been conducted rigorously, with appropriate controls, replication, and sample sizes. The conclusions must be drawn appropriately based on the data presented.

Reviewer #3: Yes

Reviewer #4: Yes

**********

3. Has the statistical analysis been performed appropriately and rigorously?

Reviewer #3: Yes

Reviewer #4: Yes

**********

4. Have the authors made all data underlying the findings in their manuscript fully available?

The PLOS Data policy requires authors to make all data underlying the findings described in their manuscript fully available without restriction, with rare exception (please refer to the Data Availability Statement in the manuscript PDF file). The data should be provided as part of the manuscript or its supporting information, or deposited to a public repository. For example, in addition to summary statistics, the data points behind means, medians and variance measures should be available. If there are restrictions on publicly sharing data—e.g. participant privacy or use of data from a third party—those must be specified.

Reviewer #3: Yes

Reviewer #4: Yes

**********

5. Is the manuscript presented in an intelligible fashion and written in standard English?

PLOS ONE does not copyedit accepted manuscripts, so the language in submitted articles must be clear, correct, and unambiguous. Any typographical or grammatical errors should be corrected at revision, so please note any specific errors here.

Reviewer #3: Yes

Reviewer #4: Yes

**********

6. Review Comments to the Author

Please use the space provided to explain your answers to the questions above. You may also include additional comments for the author, including concerns about dual publication, research ethics, or publication ethics. (Please upload your review as an attachment if it exceeds 20,000 characters)

Reviewer #3: (No Response)

Reviewer #4: All comments raised during previous reviews have been adressed. The study is clearly presented as exploratory. The design is interesting and the results are indicative of potential associations of susceptibility with specific SNPs. Authors have in particular been very cautious is stating that their results are to be confirmed on larger studies.

**********

7. PLOS authors have the option to publish the peer review history of their article (what does this mean?). If published, this will include your full peer review and any attached files.

If you choose “no”, your identity will remain anonymous but your review may still be made public.

**Do you want your identity to be public for this peer review?**  For information about this choice, including consent withdrawal, please see our Privacy Policy.

Reviewer #3: No

Reviewer #4: No

**********

# Editor report: Acceptance letter

**DOI:** 10.1371/journal.pone.0355230.r006

**Authors:** Pierre Germon

PONE-D-25-49503R2

PLOS One

Dear Dr. Nagaoka,

I'm pleased to inform you that your manuscript has been deemed suitable for publication in PLOS One. Congratulations! Your manuscript is now being handed over to our production team.

At this stage, our production department will prepare your paper for publication. This includes ensuring the following:

* All references, tables, and figures are properly cited

* All relevant supporting information is included in the manuscript submission,

* There are no issues that prevent the paper from being properly typeset

You will receive further instructions from the production team, including instructions on how to review your proof when it is ready. Please keep in mind that we are working through a large volume of accepted articles, so please give us a few days to review your paper and let you know the next and final steps.

Lastly, if your institution or institutions have a press office, please let them know about your upcoming paper now to help maximize its impact. If they'll be preparing press materials, please inform our press team within the next 48 hours. Your manuscript will remain under strict press embargo until 2 pm Eastern Time on the date of publication. For more information, please contact onepress@plos.org.

You will receive an invoice from PLOS for your publication fee after your manuscript has reached the completed accept phase. If you receive an email requesting payment before acceptance or for any other service, this may be a phishing scheme. Learn how to identify phishing emails and protect your accounts at https://explore.plos.org/phishing.

If we can help with anything else, please email us at customercare@plos.org.

Thank you for submitting your work to PLOS ONE and supporting open access.

Kind regards,

PLOS ONE Editorial Office Staff

on behalf of

Dr. Pierre Germon

Academic Editor

PLOS One
